# Modeling the Evolution of Laser-Induced Electronic Coherences with Trajectory Surface Hopping

**DOI:** 10.1021/acs.jctc.5c00531

**Published:** 2025-10-20

**Authors:** Gilbert Grell, Jesús González-Vázquez, Francisco Fernández-Villoria, Alicia Palacios, Fernando Martín

**Affiliations:** † 202533 Instituto Madrileño de Estudios Avanzados en Nanociencia (IMDEA), Madrid 28049, Spain; ‡ Departamento de Química, Módulo 13, 16722 Universidad Autónoma de Madrid, Madrid 28049, Spain; § Institute for Advanced Research in Chemical Sciences (IAdChem), 16722 Universidad Autónoma de Madrid, Madrid 28049, Spain

## Abstract

Current attosecond XUV/X-ray pulses and few-fs UV pulses, with bandwidths up to several eV, can excite molecules in a coherent superposition of electronic states. Theoretical modeling of the ensuing dynamics requires accounting for the coupled motion of electrons and nuclei to capture the subtle interplay between the initial electronic coherence, decoherence induced by nuclear motion, and additional coherences that may emerge at conical intersections. In this work, we introduce Trajectory Surface Hopping with Projected Forces and Momenta (TSH-PFM), which accounts for these effects in a numerically inexpensive way and, therefore, is particularly suited to describe molecular dynamics arising from an initial coherent superposition of electronic states. We demonstrate its performance by comparing with previously reported quantum mechanical results for the BMA­[5,5], para-xylene, and fulvene molecules by working in full dimensionality. Application of the TSH-PFM method to the glycine molecule shows that the initial electronic coherences can have a dramatic impact in the charge distribution over the molecule at the very early stages of the dynamics.

## Graphical abstract

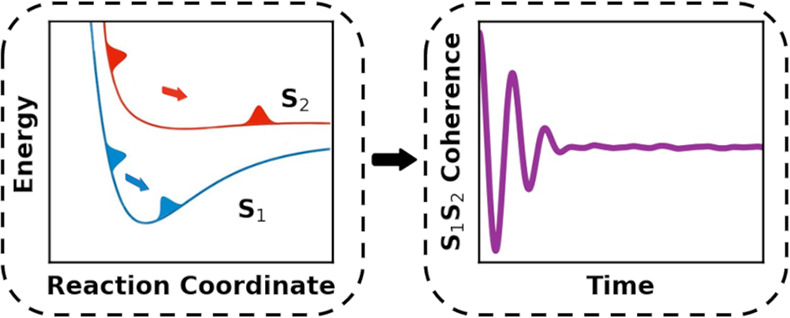

## 1 Introduction

Tully’s fewest switching trajectory surface hopping (TSH) method^1,2^ is one of the most widespread mixed quantum-classical approaches to simulate nonadiabatic molecular dynamics (see, e.g.,^3–6^ and references therein). Following the Born–Oppenheimer notion of separable atomic and electronic degrees of freedom in molecular systems, in this method the atomic positions are propagated classically on the quantum-mechanical adiabatic potential energy surface of the occupied electronic state. In regions of nonzero nonadiabatic coupling (NAC) between the currently occupied and the other electronic states, nonadiabatic transitions are included by stochastically hopping to a different electronic potential. This allows a trajectory to trespass multiple electronic potential surfaces, while following the gradient of a single, referred to as the active, electronic potential at any given time.^1,2^ A suitable ensemble of such TSH trajectories represents a discretization of the phase space explored by the full quantum dynamics, allowing for approximating quantum-mechanical observables by an ensemble integration over many trajectories.^7,8^

Despite the classical treatment of the atomic motion on the electronic quantum potential surfaces, TSH can predict the population distribution after bifurcation of a nuclear wave packet passing through a conical intersection to a reasonably good approximation.^9^ Due to its relatively low computational cost compared to quantum-mechanical treatments, TSH can be used to study nonadiabatic dynamics in rather large molecular systems (see, e.g.,^3–6,10–13^ and references therein). This has spurred the continuous development of TSH algorithms and their implementation in open-source packages such as SHARC,^14,15^ NEWTON-X,^16^ JADE,^17^ PYXAID,^18^ ANT,^19^ MLatom,^20^ and others, as well as in quantum chemistry packages such as OpenMolcas^21^ and Q-CHEM,^22^ with widespread application in femtochemistry, photophysics, photochemistry, and photobiology, to name a few.

Despite this enormous success, TSH has innate limitations due to the use of classical mechanics to describe quantum nuclear motion. In its original formulation, TSH has no access to the overlaps between the nuclear wave packets propagating in the active and inactive potentials. However, in a quantum-mechanical picture, these overlaps govern the amount of coherence between electronic states. Thus, TSH cannot account for quantum decoherence arising from the fact that nuclear wave packets evolving in different potential energy surfaces do not propagate in the same way and, therefore, may not overlap after some time.^2,23–25^ To incorporate such decoherence effects into TSH, multiple approaches have been developed to estimate the decay of the overlap between the active-potential and inactive-potential nuclear wave packets.^23–44^ These approximations employ Gaussian functions to model the nuclear wave packets, based on the fact that they keep their Gaussian character when propagated on locally harmonic potentials,^45^ thus allowing one to work with analytic expressions. Some approaches, like augmented fewest switches surface hopping (A-FSSH),^37–40^ require the explicit evaluation of gradients for both, active and inactive potentials. However, to keep computational cost at the level of conventional TSH simulations, most widely used methods circumvent the evaluation of gradients in potentials other than the active ones.^31–36,42^ In particular, the energy-based decoherence correction (EDC) approach introduced by Granucci and Persico,^35^ based on previous work by Zhu, Truhlar and co-workers,^32,33^ represents a strikingly simple approximation, in which the change of the nuclear overlap is translated into a decoherence rate that is solely determined by the active-inactive potential energy difference and the kinetic energy. EDC became, perhaps, the most commonly used and widely available decoherence correction due to its low numerical cost and remarkable performance for simulating a wide range of dynamical processes.^5,15,16,19,20,46,47^

As a consequence of the experimental developments in the field of ultrafast molecular dynamics with the advent of femtosecond chemistry,^48^ TSH and related methods were designed to simulate nonadiabatic excited state dynamics^49^ arising from a narrow distribution of total energies in the ensemble. Such may be obtained by excitations with continuous-wave, and most (narrow-band) femtosecond laser systems. In the case of such laser-excitations, one usually has just a single initially populated electronic potential, albeit it may contain a vibrational coherence. After such an excitation, electronic coherences, which we target in this work, can only arise at NAC regions where the initial wave packet splits onto several electronic potential surfaces. Since energy conservation determines the kinetic energies of the wave packets created by, e.g., passing a conical intersection, the EDC and related approaches,^32,33,35^ appear well-suited for this situation.

With the advent of attosecond science,^50^ XUV and soft X-ray pulses with duration of a few hundreds attoseconds are available.^50–52^ Similarly short pulses, but much more intense and extending up to the hard X-ray spectral region, have also been recently produced in large-scale X-ray free-electron laser facilities.^53,54^ The attosecond is the natural time scale of electronic motion. Thus, not surprisingly, attosecond pulses are currently being used to investigate electron dynamics in matter, in particular, molecules.^55–70^ The bandwidth associated with such pulses can be as large as a few tens of eV, which is larger than the energy spacings between consecutive electronic states. As a consequence, a coherent superposition of electronic states is produced, which evolves with characteristic periods ranging from the subfemtosecond to the few femtosecond time scale.

XUV and X-ray radiation inevitably leads to ionization of any molecule after absorption of a single photon. So, experiments performed with XUV and X-ray attosecond pulses only allow for the investigation of electron dynamics in molecular cations. However, very recently, UV pulses as short as 2–3 fs have become available,^71,72^ which can also lead to a coherent superposition of electronic states, but now in neutral molecules. Their short duration ensures the necessary time resolution to investigate and eventually control electron dynamics arising from laser-induced coherent superpositions of electronic states in neutral molecules, which is the ultimate goal of the emerging field of attochemistry.^60,68,73^

The theoretical description of nonadiabatic molecular dynamics after excitation with broad-band few-femtosecond or attosecond pulses is necessary, as it introduces decoherence and dephasing, thus determining the evolution and survival of the initial coherence.^74–76^ Furthermore, the electronic populations can be altered considerably by nonadiabatic effects even at very early times. Such simulations require methods able to treat an initial coherent superposition of electronic states that may be separated by several eV up to a few tens of eV, leading to a very wide distribution of total energies, thus exceeding the conventional realm of TSH simulations. Therefore, it is not clear whether one can apply TSH for such situations, and, if so, which decoherence correction one should use, and how observables can be evaluated.^8^ Since the target is ultimately the evaluation of dynamic observables that probe the electronic coherence, the decoherence correction needs to be chosen with great care. It may impact coherence-sensitive observables stronger than conventional observables that depend solely on the population distribution.

In this work, we present a novel decoherence correction to the TSH methodology that has been especially designed to describe the coupled electron–nuclear dynamics that arises from an initial coherent superposition of electronic states. The novelty of the approach, hereafter called “projected forces and momenta” (PFM) decoherence correction, is that it accounts for the effect of the forces and momenta on the inactive potentials projected along the direction of the mass-weighted velocity in the active potential. These auxiliary forces and momenta do not require the evaluation of explicit gradients in the inactive potentials, i.e., they do not add to the numerical cost of conventional fewest-switching TSH simulations.

We apply the PFM decoherence correction to fewest-switches TSH simulations (TSH-PFM) of the nonadiabatic dynamics in various organic molecules with and without an initial electronic coherence. We show that TSH-PFM simulations predict a survival of initial electronic coherences similar to fewest-switches TSH simulations with no decoherence correction (TSH-ND) and, for the cases where such results are available in the literature,^75^ in reasonable agreement with the direct dynamics variational multiconfigurational Gaussian (DD-vMCG) method^77,78^ that employs full-dimensional coupled quantum trajectories. At longer times and without an initial coherence we find that TSH-PFM simulations predict essentially the same dynamics as one obtains with the widely used energy-based decoherence correction (TSH-EDC). TSH-PFM simulations may thus provide a comparatively inexpensive tool to investigate the dynamics that follows excitation by broadband laser pulses.

The paper is organized as follows. In the theory section, we derive the PFM decoherence correction starting from a seminal work by Jasper and Truhlar^34^ and discuss its physical interpretation. Subsequently we detail the TSH-PFM algorithm implemented in a locally modified version of the SHARC package.^14,79^ In the results section we first scrutinize details of the auxiliary momentum propagation for the PFM decoherence correction, by comparing, for instance, to full-quantum results for the dynamics in the IBr molecule.^14^ We then benchmark TSH-PFM by comparing with DD-vMCG results for the dynamics following an initial coherence in the modified bismethylene-adamantane (BMA­[5,5]) and para-xylene molecules^75^ and against ab initio multiple spawning (AIMS)^80–82^ results for the dynamics in fulvene, a molecular representation of Tully’s model III.^83^ We also compare with TSH-ND and TSH-EDC calculations in all cases, to elucidate the performance of the established TSH approaches under the paradigm of broad-band excitations. This comparison is further extended to the glycine molecule, for which no quantum calculations have been reported so far for an initial coherent superposition of electronic states. For glycine, we discuss the possible implications of these initial coherences on the charge dynamics by analyzing the evolution of the molecular dipole. We end with a summary of the main conclusions of this work.

## 2 Theory

### 2.1 Electronic Coherence and Decoherence in Molecular Dynamics

In a full quantum mechanical treatment, the molecular wave function can be written in terms of a Born–Huang expansion^84,85^ over the adiabatic electronic eigenstates, Ψ_*i*_(***r***, ***R***), depending parametrically on the 3*N*_*A*_ nuclear coordinates ***R***,

$$\Psi (r,R,t)=\sum_{i}^{\infty }\Psi_{i}(r,R){\mathrm{\chi ̃}}_{i}(R,t)\equiv \sum_{i}^{\infty }\Psi_{i}(r,R)c_{i}(t)\chi_{i}(R,t)$$ 1

In this expression, each electronic state is multiplied by the nuclear wave functions, χ̃_*i*_(***R***, *t*), obtained by solving the fully coupled nuclear time-dependent Schrödinger equation (TDSE) for all populated electronic potential energy surfaces. For convenience, they have been formally renormalized by introduction of the coefficients *c*_*i*_(*t*), such that

$$\int dR\;\chi_{i}^{*}(R,t)\chi_{i}(R,t)=1$$ 2

$$\sum_{i}|c_{i}(t){|}^{2}=1$$ 3

In this representation, the electronic density matrix elements at a given molecular geometry R are given by

$$\rho_{ij}(R,t)=c_{i}(t)c_{j}^{*}(t)\chi_{i}(R,t)\chi_{j}^{*}(R,t)$$ 4

which, after integration over ***R***, can be written as

$$\rho_{ij}(t)=c_{i}(t)c_{j}^{*}(t)\int dR\;\chi_{i}(R,t)\chi_{j}^{*}(R,t)\equiv c_{i}(t)c_{j}^{*}(t)S_{ij}^{*}(t)$$ 5

When ρ_*ij*_(***R***,*t*) is localized in a small volume around the position ***R***_0_, its integral, ρ_*ij*_(*t*), can be interpreted as the purely electronic density matrix element at ***R***_0_, if the respective electronic states, Ψ_*i*_(***r***, ***R***), Ψ_*j*_(***r***, ***R***), vary only weakly with ***R*** in that region. The diagonal elements correspond to the electronic populations, ρ_*ii*_(*t*) = |*c*_*i*_(*t*)|^2^, and the off-diagonal ones, ρ_*i* ≠ *j*_(*t*), are the electronic coherences, the magnitude of which critically depends on the nuclear wave function overlap, *S*_ij_^*^(*t*).

In the TSH method,^1,2^ the atomic positions are propagated according to the classical equations of motion along the quantum-mechanical electronic potential surfaces. For every initial set of positions and momenta, {***R*** (0), ***P*** (0)}, this yields a semiclassical trajectory ***R*** (*t*), along which the electronic wave function is defined as

$$\Psi (r,R(t),t)=\sum_{i}c_{i}(t)\Psi_{i}(r,R(t))$$ 6

As TSH does not contain an explicit description of the nuclear wave function, the electronic density matrix elements are simply given by,

$$\rho_{ij}^{\mathrm{TSH}}(t)=c_{i}(t)c_{j}^{*}(t)$$ 7

Consequently, electronic decoherence due to the decay of the nuclear overlap between two wave packets traveling on different electronic potentials is not accounted for and artificial long-lived coherent oscillations and nonphysical interferences may arise.^2,23–25^

### 2.2 Derivation of the Decoherence Rate

The use of Gaussian wave packets to assign quantum character to classical trajectories in semiclassical methods for solving the molecular TDSE has been introduced by Heller.^45,86–88^ This was based on the observation that initially Gaussian wave packets retain their Gaussian character when propagated along locally quadratic potentials, although they may spread and acquire a phase, while their position and momentum centroids follow classical equations of motion (EOMs). The extension to arbitrary potential surfaces holds for narrow wave packets on sufficiently smooth potential surfaces. In this context, the frozen Gaussian approximation (FGA)^87^ was introduced as a cost-effective approach to model the dynamics. Since then it has been at the center of many methods to model nonadiabatic molecular dynamics by explicitly incorporating nuclear wave functions, such as direct dynamics variational multiconfigurational Gaussian (DD-vMCG),^77,78^ ab initio multiple spawning (AIMS),^80–82^ and multiconfigurational Ehrenfest (MCE),^89^ to name a few.

Dressing classical trajectories with frozen Gaussian wave packets has been instrumental to show that the decoherence rate between two electronic potentials depends on the respective force difference.^23,24,26,28,34^ This ansatz has since been used as the starting point to derive a manifold of decoherence corrections for semiclassical methods.^24,28–30,34,36,38–42,44^ Here we follow a similar approach and, for a molecule containing *N*_*A*_ atoms, use a multidimensional frozen Gaussian to approximate the nuclear wave function along a trajectory in the electronic potential *i*. Employing atomic units here and throughout the manuscript, one has,

$$\chi_{i}(R,t)=\frac{e^{-1/4(R-R_{i}(t){)}^{T}\Sigma_{i}^{-1}(R-R_{i}(t))}}{((2\pi {)}^{3N_{A}}\det\;\Sigma_{i}{)}^{1/4}}e^{iP_{i}(t{)}^{T}(R-R_{i}(t))}e^{i\Lambda_{i}(t)}$$ 8

where ***R***_*i*_(*t*) and ***P***_*i*_(*t*) are the position and momentum centroids classically propagated in a given electronic potential. The inverse covariance matrix, **Σ**_*i*_^–1^, couples the 3*N*_*A*_-dimensional Cartesian positions, ***R***, while Λ_*i*_(*t*) is a time-dependent phase accumulated during the propagation. We now introduce mass-weighted coordinates,^90^ in order to take advantage of the fact that mass-weighted velocities and momenta, as well as mass-weighted accelerations and forces are the same,

$$Q=M^{1/2}R$$ 9

$$\mathrm{Q̇}=M^{1/2}\dot{R}=M^{-1/2}P$$ 10

$$\mathrm{Q̈}=M^{1/2}\ddot{R}=M^{-1/2}F$$ 11

where dots indicate time derivatives, e.g., ***Q̇*** = ∂_*t*_ ***Q*** , and ***M*** is the mass matrix in Cartesian coordinates. One has the nuclear wave function, eq 8, in mass-weighted coordinates,

$$\chi_{i}(Q,t)={\left(\frac{\det\;\Omega }{\pi^{3N_{A}}}\right)}^{1/4}e^{-1/2(Q-Q_{i}(t){)}^{T}\Omega \;(Q-Q_{i}(t))}\times e^{i{\mathrm{Q̇}}_{i}^{T}(t)(Q-Q_{i}(t))}e^{i\Lambda_{i}(t)}$$ 12

where we have introduced the frequency matrix,

$$\Omega =\frac{1}{2}M^{-1/2}\Sigma^{-1}M^{-1/2}$$ 13

with the assumption that the covariance matrix is the same for all electronic potentials. This follows previous works by, e.g., Rossky and co-workers^24,28^ and Jasper and Truhlar^34^ and corresponds to an almost instantaneous population of the electronic states, e.g., by a very short pulse. The (real and symmetric) frequency matrix **Ω** is positive semidefinite with *N*_*D*_ = 3*N*_*A*_–6 (*N*_*D*_ = 3*N*_*A*_–5 for linear molecules) nonzero eigenvalues corresponding to the normal-mode frequencies ω_1_, ···, ω_*N*_*D*__ > 0 along the normal mode coordinates defined by the corresponding eigenvectors arranged as columns of the orthogonal matrix ***V*** = (***v***_1_··· ***v***_*N*_*D*__).

$$\Omega =V\mathrm{diag}\{\overset{>0}{\overset{︷}{\omega_{1},...,\omega_{N_{D}}}},\overset{0}{\overset{︷}{\omega_{N_{D}+1},...,\omega_{3N_{A}}}}\}V^{T}$$ 14

The remaining, zero frequencies, ω_β_ = 0 for β = *N*_*D*_ + 1, ···,3*N*_*A*_, correspond to the rotational and translational degrees of freedom (DOFs). They do not contribute to the dynamics, and are removed by decomposing the frequency matrix and introducing normal mode coordinates,

$$\Omega =\sum_{\alpha =1}^{N_{D}}\omega_{\alpha }v_{\alpha }v_{\alpha }^{T}$$ 15

$$q_{\alpha }=v_{\alpha }^{T}Q$$ 16

and analogous for the velocities. One obtains the decoupled wave function in normal mode coordinates, denoted by small letters in the following,

$$\chi_{i}(q,t)=\left[\prod_{\alpha =1}^{N_{D}}{\left(\frac{\omega_{\alpha }}{\pi }\right)}^{1/4}e^{-\omega_{\alpha }/2(q_{\alpha }-q_{i,\alpha }(t){)}^{2}+i{\mathrm{q̇}}_{i,\alpha }(t)(q_{\alpha }-q_{i,\alpha }(t))}\right]\times e^{i\Lambda_{i}(t)}$$ 17

Note that the normal-mode decomposition is only accurate in the harmonic region of the electronic potential, for which it has been evaluated.^45,86,87^ It is employed herein as the decoupling simplifies the following derivation considerably, however, our final result is independent of the normal mode coordinates.

The first steps of our derivation until eq 25 closely follow the work of Jasper and Truhlar,^34^ differing only in the consideration of a multidimensional nuclear wave function in mass-weighted normal mode coordinates. Neglecting all phase terms, the absolute magnitude of the overlap between two nuclear wave functions given by eq 17, propagated on electronic potentials *i* and *j*, can be written as

$$|S_{ij}(t)|=e^{-K_{ij}(t)}$$ 18

where *K*_*ij*_(*t*) is given as the sum over the contributions from all DOF in terms of the normal mode position and velocity differences between the respective electronic potentials, *q*_*ij*, α_(*t*) = *q*_*i*, α_(*t*)–*q*_*j*, α_(*t*), and analogous for *q̇*_*ij*, α_(*t*),

$$K_{ij}(t)=\sum_{\alpha =1}^{N_{D}}K_{ij,\alpha }(t)$$ 19

$$K_{ij,\alpha }(t)=\frac{\omega_{\alpha }q_{ij,\alpha }^{2}(t)}{4}+\frac{{\mathrm{q̇}}_{ij,\alpha }^{2}(t)}{4\omega_{\alpha }}$$ 20

Expanding the decay exponent up to first order in time,^34^ one obtains for small Δ*t*,

$$|S_{ij}(t+\Delta t)|\approx |S_{ij}(t)|e^{-k_{ij}(t)\Delta t}$$ 21

where *k*_*ij*_(*t*) is the first-order decoherence rate in the time interval [*t*, *t* + Δ*t*], given by

$$k_{ij}(t)=\partial_{t}K_{ij}(t)=\overset{N_{D}}{\underset{\alpha =1}{\sum }}\overset{k_{ij,\alpha }(t)}{\overset{︷}{\partial_{t}K_{ij,\alpha }(t)}}$$ 22

Dropping explicit time arguments, the decoherence rate for each DOF, α, reads,

$$k_{ij,\alpha }=\frac{\omega_{\alpha }}{2}q_{ij,\alpha }{\dot{q}}_{ij,\alpha }+\frac{1}{2\omega_{\alpha }}{\dot{q}}_{ij,\alpha }{\ddot{q}}_{ij,\alpha }$$ 23

Note the appearance of the normal-mode acceleration differences, *q̈*_*ij*, α_, corresponding to the mass-weighted force-differences along the respective coordinate. Following ref 34 in choosing the position differences, *q*_*ij*,α_, such as to maximize the decoherence by requiring that **0** = ∇_***q***_*ij*__(∂_*t*_|*S*_*ij*_|), yields

$$q_{ij,\alpha }^{\pm }=-\frac{{\ddot{q}}_{ij,\alpha }}{2\omega_{\alpha }^{2}}\pm \sqrt{\frac{{\mathrm{q̈}}_{ij,\alpha }^{2}}{4\omega_{\alpha }^{4}}+\frac{2}{\omega_{\alpha }}}$$ 24

To keep the decoherence rate greater than zero, one selects *k*_*ij*,α_(*q*_*ij*,α_^+^), for *q̇*_*ij*, α_ ≥ 0 and *k*_*ij*,α_(*q*_*ij*,α_^–^), for *q̇*_*ij*,α_ < 0 when inserting eq 24 into eq 23, assuming that the wave packet in the state with the larger velocity has traveled farther along ***v***_α_. This yields the multidimensional generalization of the previously reported one-dimensional result^34^ in normal-mode coordinates and frequencies,

$$k_{ij}^{\mathrm{FM}}=\overset{N_{D}}{\underset{\alpha =1}{\sum }}\frac{1}{4\omega_{\alpha }}({\overset{˙}{q}}_{ij,\alpha }{\overset{¨}{q}}_{ij,\alpha }+|{\overset{˙}{q}}_{ij,\alpha }|\sqrt{{\overset{¨}{q}}_{ij,\alpha }^{2}+8\omega_{\alpha }^{3}})$$ 25

which we will in the following refer to as the forces and momenta (FM) decoherence rate. Note that the special case of a one-dimensional system, e.g., a diatomic molecule with reduced mass, μ, bond-length momenta and forces, *p*_*i*_ and *f*_*i*_, and a width of the wave function, σ, is obtained as,^34^

$$k_{ij}^{\mathrm{FM}-1D}=\frac{\sigma^{2}}{2}\left(p_{ij}\;f_{ij}+|p_{ij}|\sqrt{f_{ij}^{2}+\frac{1}{\mu^{2}\sigma^{6}}}\right)$$ 26

The respective one-dimensional mass-weighted formula, eq 25, is obtained with the transformations ${\dot{q}}_{ij}=\mu^{-1/2}p_{ij}$ , ${\ddot{q}}_{ij}=\mu^{-1/2}f_{ij}$ , and, $\omega =\frac{1}{2{\mu \sigma }^{2}}.$ Hereafter, our derivation substantially deviates from the original work,^34^ as we keep the frequency (or the variance in 1D systems), as an explicit parameter and seek a new set of approximations in the multidimensional case.

If the system has a single DOF, the velocities (momenta) and accelerations (forces) *q̇*_*ij*,α_ and *q̈*_*ij*,α_ can be evaluated easily and eqs 25 and 26 can be directly used. For the multidimensional case, *N*_*D*_ > 1, evaluation of the decoherence rate, eq 25, requires the full velocity (momentum) and acceleration (force) vectors in all populated electronic potentials. The required calculation of gradients in all populated electronic potentials with high-level electronic structure methods, such as complete active space second-order perturbation theory (CASPT2), is, however, computationally expensive even for comparatively small systems. Due to this, one usually evaluates only the active-potential force and disregards all inactive-potential gradients in the same way as it is common practice to approximate the NAC terms by the inexpensive numerical time derivative of the overlap.^27^ However, this makes the FM-decoherence correction according to eq 25 inaccessible, as well as e.g., A-FSSH.^38,40^ A formal disadvantage of the FM decoherence rate is that it depends explicitly on a normal-mode coordinate system corresponding to a specific region of a single electronic potential. This may become problematic if the involved potentials surfaces have different characteristics, as well as if the wave packet leaves the initial potential region. In what follows we circumvent these problems and only require the active-potential forces at every nuclear time step.

We first assume that the frequency is the same in all DOFs. It is taken as the geometrical average over the nonzero frequencies,

$$\omega ={\left(\prod_{\alpha =1}^{N_{D}}\omega_{\alpha }\right)}^{1/N_{D}}$$ 27

As the frequencies correspond to inverse variances along the normal modes, this choice conserves the original volume of the variance ellipse of the wave function, eq 12.

To avoid ambiguity in practical calculations, we suggest to estimate the frequency parameter from the (harmonic) normal-mode frequencies, which can be obtained easily with most quantum-chemistry packages for the electronic-structure method of choice. If a different treatment is desired, one can, in principle, evaluate the covariance matrix directly for a given ensemble of initial conditions, and use eqs 13 and 14, to obtain the frequencies. Finally, ω can also be interpreted as a free parameter of the decoherence model.

Assuming that ω is known, a general upper bound to eq 25 is obtained by using $\sqrt{a^{2}+b^{2}}\leq |a|+|b|$ , without assumptions on the ratio of *a* and *b*,

$$k_{ij}^{\mathrm{FM}}\leq \frac{1}{4\omega }\left(\sum_{\alpha =1}^{N_{D}}{\dot{q}}_{ij,\alpha }{\ddot{q}}_{ij,\alpha }+|{\dot{q}}_{ij,\alpha }||{\ddot{q}}_{ij,\alpha }|+\sqrt{8\omega^{3}}|{\dot{q}}_{ij,\alpha }|\right)$$ 28

By introducing the notation for vectors of absolute values,

$$[x]=(|x_{1}|,\cdot \cdot \cdot ,|x_{N_{D}}|{)}^{T}$$ 29

we may rewrite all component sums in eq 28 as scalar products,

$$k_{ij}^{\mathrm{FM}}\leq \frac{1}{4\omega }({\dot{q}}_{ij}^{T}{\ddot{q}}_{ij}+[\dot{q}{]}_{ij}^{T}[\ddot{q}{]}_{ij}+\sqrt{8\omega^{3}}[\dot{q}{]}_{ij}^{T}1^{N_{D}})$$ 30

Here, $1^{N_{D}}=(1,\cdot \cdot \cdot ,1{)}^{T}\in R^{N_{D}}$ is the *N*_*D*_-dimensional 1-vector. Now, we replace the scalar products with their upper bound, ***x*** ^*T*^ ***y*** ≤ | ***x*** ∥ ***y*** |, to remove the vector character from the expression. Using |[***x***]| = | ***x*** | and $|1^{N_{D}}|=\sqrt{N_{D}}$ , one finds an upper bound to eq 30,

kijFM≤12ω|q˙ij||q¨ij|+|q˙ij|NDω2(31)≤12ω|Q˙ij||Q¨ij|+|Q˙ij|NDω2(32) 31

In eq 32 we remove the dependence on the normal-mode transformation, since, under the given approximations, the length of the velocity and acceleration vectors may not depend on the coordinate system. The r.h.s. of eq 32 represents an upper bound to the decoherence rate given initially in eq 22 if the nuclear wave functions in all electronic potentials are well-described by a product of *N*_*D*_ decoupled frozen Gaussians with the same variance, $\frac{1}{2\omega }$ , in all DOFs.

To evaluate decoherence rates with eq 32 in practice, one needs suitable approximations for the absolute values of the force, momentum, and velocity differences. Such can be obtained from the time derivative of the potential energy, $E_{i}.$ In Cartesian coordinates this yields the projection of the force, ***F***_*i*_(*t*), onto the trajectory velocity, ***Ṙ*** (*t*), and in mass-weighted coordinates, respectively, the projection of the mass-weighted acceleration, ***Q̈***_*i*_(*t*), onto the velocity, ***Q̇*** (*t*),

E˙i(t)=∇RTEi(R(t))︷−FiT(t)Ṙ(t)(33)=∇QTEi(Q(t))︷−Q̈iT(t)Q̇(t)(34) 33

Dividing eq 34 by the norm of the mass-weighted trajectory velocity, *v*_*m*_(*t*) = | ***Q̇*** (*t*)|, yields the projection of the acceleration onto the direction of the trajectory velocity, ***e***_*vm*_(*t*) = ***Q̇*** (*t*)/*v*_*m*_(*t*),

$$F_{m,i}^{v}(t)={\mathrm{Q̈}}_{i}^{T}(t)e_{vm}(t)=-\frac{{\dot{E}}_{i}(t)}{v_{m}(t)}$$ 35

which we refer to as the mass-weighted trajectory-velocity-projected (TVP) force. It is used to propagate the corresponding mass-weighted TVP momenta, for which one has

$$P_{m,i}^{v}(t)={\dot{Q}}_{i}^{T}(t)e_{vm}(t)$$ 36

$${Ṗ}_{m,i}^{v}(t)=F_{m,i}^{v}(t)+{\dot{Q}}_{i}^{T}(t){\dot{e}}_{vm}(t)$$ 37

The second term in eq 37 corresponds to the change of the mass-weighted trajectory velocity direction projected onto the inactive-potential velocity. Its calculation requires the previously discarded evaluation of inactive-potential gradients, so that it is set to zero, i.e., we assume a collinear change of the mass-weighted trajectory velocity, ***ė***_*vm*_(*t*) ≈ 0. Integrating eq 37 with the left-sided sum rule one obtains the mass-weighted TVP momentum propagator in collinear-trajectory-velocity-change approximation,

$$P_{m,i}^{v,\mathrm{col}}(t+\Delta t)\approx P_{m,i}^{v,\mathrm{col}}(t)+F_{m,i}^{v}(t)\Delta t$$ 38

The absolute mass-weighted velocity and acceleration differences in eq 32 are then approximated in terms of their TVP counterparts. If ϑ is the angle between the velocity direction and mass-weighted acceleration difference, ***e***_*vm*_ and ***Q̈*** _*ij*_, one has

$$\left|F_{m,ij}^{v}|=|{\ddot{Q}}_{ij}||\cos\;\vartheta \right|$$ 39

To remove the angular dependence, we assume that all projection angles between the mass-weighted trajectory velocity and acceleration-difference vectors are equally probable in the multidimensional case and replace the |cos ϑ| term by its average, 2/π,

$$\left|F_{m,ij}^{v}|\approx |{\ddot{Q}}_{ij}|\frac{1}{2\pi }\int_{0}^{2\pi }|\cos\;\vartheta |d\vartheta =\frac{2}{\pi }|{\ddot{Q}}_{ij}\right|$$ 40

Although this may be an impactful approximation in highly dimensional systems, it is adopted here as a straightforward solution to remove the dependence on the projection angle. Analogous equations hold for the mass-weighted TVP momentum difference, so that the absolute values of the mass-weighted velocity and acceleration differences in eq 32 may be approximated as,

$$\left|{\ddot{Q}}_{ij}|\approx \frac{\pi }{2}|F_{m,ij}^{v}\right|$$ 41

$$\left|{\dot{Q}}_{ij}|\approx \frac{\pi }{2}|P_{m,ij}^{v,\mathrm{col}}\right|$$ 42

Inserting eqs 41 and 42 into eq 32 yields the decoherence rate in terms of the mass-weighted TVP forces and momenta,

$$k_{ij}^{\mathrm{PFM}}=\frac{\pi^{2}}{8\omega }|P_{m,ij}^{v,\mathrm{col}}||F_{m,ij}^{v}|+|P_{m,ij}^{v,\mathrm{col}}|\;\sqrt{\frac{\pi^{2}N_{D}\;\omega }{8}}$$ 43

In the following, this result will be referred to as the PFM decoherence rate.

### 2.3 Inclusion of Decoherence into TSH

In the fewest-switching TSH approach, the adiabatic electronic coefficients are propagated along a trajectory ***R*** (*t*) according to the electronic TDSE,^1,2^

$${\overset{˙}{c}}_{i}(t)=-\;\left[{iE}_{i}(R(t))c_{i}(t)+\underset{j}{\sum }K_{ij}(R(t))c_{j}(t)\right]$$ 44

Here, $E_{i}$ ­(***R*** (*t*)) are the adiabatic electronic potential energies and the NAC is defined as the time-derivative coupling,

Kij(R(t))=∫drΨi*(r,R(t))∂tΨj(r,R(t))(45)=[∫drΨi*(r,R(t))∇RTΨj(r,R(t))]︸dijR˙(t)(46) 45

To avoid the numerically cumbersome evaluation of the NAC vectors, ***d***_*ij*_, we approximate the time-derivative coupling by the finite difference,^27^

$$K_{ij}(R(t))\approx \frac{\delta_{ij}-\langle \Psi_{i}(R(t))|\Psi_{j}(R(t-\Delta t))\rangle }{\Delta t}$$ 47

Albeit this approximation allows only for isotropic velocity rescaling to conserve the energy after surface hops, see discussion in Section 2.5, it is commonly employed for its computational simplicity.^6,15,16,47,91^

The FM and PFM decoherence rates, eqs 26 and 43, represent first-order decay rates of the overlap between two nuclear wave packets moving on different electronic potentials *i*, *j*, see eq 21, in line with the EDC and other well-established methods to include decoherence into nonadiabatic dynamics simulations.^24,25,32–35,37,92^ Following previous work,^32,33,35^ we incorporate decoherence by modifying the electronic coefficients at the end of each TDSE integration time step after the evaluation of the stochastic surface-hopping procedure. The inactive-potential coefficients are damped with the first-order decay of the active–inactive nuclear overlap, |*S*_*ia*_(*t*)|, see eq 21, while the imparted loss of inactive-potential population is compensated by scaling the active-potential coefficients accordingly,

$$c_{i}(t)=c_{i}(t)e^{-k_{ia}(t-\Delta t)\Delta t}$$ 48

$$c_{a}\left(t\right)=\frac{c_{a}\left(t\right)}{\left|c_{a}\left(t\right)\right|}\sqrt{1-\underset{i\neq a}{\sum }{\left|c_{i}\left(t\right)\right|}^{2}}$$ 49

Here and in the remaining part of the theory section, the indices, *a*, *i*, and *j* indicate the active, inactive, and all electronic potentials, respectively. The decoherence correction thus ensures that after a certain time and far from a NAC region only the active potential is populated, improving the internal consistency of TSH simulations.^35,93,94^

### 2.4 Evaluation of Observables and Internal Consistency

An ensemble of ν = 1, ···, *N*_traj_ TSH-trajectories is internally consistent if, for every electronic potential, the fraction of trajectories propagating along its gradient, Π_*j*_(*t*), agrees with the quantum population obtained by averaging the adiabatic electronic populations over all trajectories, ρ_*jj*_(*t*),^93^

$$\overset{\rho_{jj}(t)}{\overset{︷}{\frac{1}{N_{\mathrm{traj}}}\overset{N_{\mathrm{traj}}}{\underset{\nu =1}{\sum }}|c_{j}^{\nu }(t){|}^{2}}}\approx \overset{\Pi_{j}(t)}{\overset{︷}{\frac{N_{j}(t)}{N_{\mathrm{traj}}}}}$$ 50

Internal consistency plays a critical role for the credibility of observables evaluated from an ensemble of TSH trajectories. One averages over the observables evaluated along the trajectories accounting for (i) only the active potential, disregarding the actual electronic coefficients, or (ii) the full electronic wave function.^7,8^ Approach (i), disregarding the electronic coefficients, has been identified as the more accurate variant, in particular without decoherence correction,^35,93,95–97^ however, it is insensitive to electronic coherence effects. Since the goal of this work is to describe electronic coherences, we resort to approach (ii). It is justified by the notion that proper account of decoherence for the system at hand establishes internal consistency,^35,36,42,47,93,94,98^ which then ensures the equivalence of (i) and (ii) for a given ensemble of trajectories. However, one must keep in mind that the choice of the proper decoherence correction may be in itself difficult while also affecting the result.^94^

### 2.5 Evaluation of Auxiliary Forces and Momenta

We are going to employ the PFM decoherence rate, eq 43, for *N*_*D*_ > 1-dimensional systems, while the FM decoherence, eq 26, will be used for a diatomic molecule, where the approximations made to obtain the PFM decoherence rates loose their meaning. The decoherence rates depend on the respective actual (FM) and mass-weighted TVP (PFM) forces and momenta for the active and inactive electronic potentials. They are denoted in this section irrespectively as auxiliary forces, *F* _*j*_^aux^(*t*), and momenta, *P* _*j*_^aux^(*t*), since they satisfy the same equations. The auxiliary forces are instantaneous properties, see eq 35, which are in practice evaluated by using the numerical forward time derivative of the potential energy,

$$F_{j}^{\mathrm{aux}}(t)=\{\begin{array}{ll} -\frac{E_{j}(t+\Delta t)-E_{j}(t)}{v(t)\Delta t} & ,\;\mathrm{if}\;|v(t)|\geq \kappa \\ 0 & ,\;\mathrm{if}\;|v(t)|<\kappa \end{array}$$ 51

Here, *v*(*t*), denotes the absolute mass-weighted velocity, | ***Q̇*** (*t*)|, for the mass-weighted TVP forces, eq 35, and the bond-length velocity, *ṙ_b_* (*t*), in diatomics. To obtain a numerically stable propagation, the auxiliary forces are set to zero, whenever the absolute value of the velocity is smaller than a threshold κ, for which we choose κ = 10^–9^ in our implementation. In practice this is mostly only relevant when starting from zero initial velocity.

The auxiliary momenta are obtained as follows. We assume that the active potential and those inactive potentials with populations above a small threshold, |*c*_*i*_(*t*)|^2^ ≥ η, each carry a single nuclear wave packet with an independent momentum propagation. Their initial auxiliary momenta, *P*_*j*_^aux^(0), are obtained from the initial condition,

$$P_{j}^{\mathrm{aux}}(0)=P^{\mathrm{aux}}(0)$$ 52

$$P^{\mathrm{aux}}(0)=\{\begin{array}{ll} \mathrm{Q̇}(0{)}^{T}e_{vm}(0) & ,\;\mathrm{if}\;N_{D}>1\\ \mu {\overset{˙}{r}}_{b}(0) & ,\;\mathrm{if}\;\mathrm{diatomic} \end{array}$$ 53

The variants of eq 53 correspond to the mass-weighted TVP momentum for the PFM decoherence, i.e., multidimensional nuclear dynamics, and actual momentum for the FM decoherence, i.e., one-dimensional dynamics of a diatomic molecule with the reduced mass μ. As a consequence of eq 52, the FM and PFM decoherence rates, eqs 26 and 43, vanish at *t* = 0 for all inactive potentials with initial above-threshold population, |*c*_*i*_(0)|^2^ ≥ η.

At *t* > 0, the auxiliary momenta of the active potential and those inactive potentials carrying population above a small threshold, |*c*_*i*_(*t*)|^2^ ≥ η, are propagated according to the classical EOM,

$$P_{a(t+\Delta t)}^{\mathrm{aux}}(t+\Delta t)=P_{a(t)}^{\mathrm{aux}}(t)+F_{a(t+\Delta t)}^{\mathrm{aux}}(t)\Delta t$$ 54

$$P_{i}^{\mathrm{aux}}(t+\Delta t)=P_{i}^{\mathrm{aux}}(t)+F_{i}^{\mathrm{aux}}(t)\Delta t$$ 55

For the active potential, *a*(*t* + Δ*t*), the propagation continues from the auxiliary momentum in the previous active potential, *a*(*t*), keeping the continuity of the active-potential properties in case of a surface hop, *a*(*t* + Δ*t*) ≠ *a*(*t*). Note that we assume the collinear-trajectory-velocity-change approximation also for the active-potential mass-weighted TVP momenta for the PFM decoherence, see the discussion around eq 38, although the accurate quantities are available. This is done to keep consistency with the inactive-potential TVP momenta.

Equation 54 mimicks the propagation of the trajectory velocity along the active potential.^2^ Following the analogy, the active-potential auxiliary momenta are modified in the same way as the trajectory velocity to conserve the total energy in case of a surface hop. We employ isotropic velocity rescaling to avoid the expensive calculation of the full NAC vectors,^27^ which would be necessary for the size-consistent velocity modification along the NAC direction,^2,16,40,47,91^

$$P_{a(t+\Delta t)}^{\mathrm{aux}}(t+\Delta t)=P_{a(t+\Delta t)}^{\mathrm{aux}}(t+\Delta t)\sqrt{1-\frac{\Delta E}{T(t+\Delta t)}}$$ 56

*T*(*t* + Δ*t*) is the kinetic energy before velocity rescaling and ΔE the total-energy difference,

$$\Delta E=E_{a(t+\Delta t)}(t+\Delta t)+T(t+\Delta t)-(E_{a(t)}(t)+T(t))$$ 57

At this point one has ΔE ≤ *T*(*t* + Δ*t*), since hops to potentials that would impose ΔE > *T*(*t* + Δ*t*) are frustrated, i.e., not carried out, since there is not enough kinetic energy to cover the energy gap. Note that we do not reverse the velocity direction in case of frustrated hops. Details on the different strategies to deal with energy conservation and the velocity direction in the case of (frustrated) surface hops, are discussed in the literature,^2,6,27,40,47,99^ and the respective sources therein.

Subthreshold populated inactive potentials, |*c*_*i*_(*t*)|^2^ < η, are, in turn, regarded as not containing a nuclear wave packet and their auxiliary momenta are set to the active-potential value, rescaled to conserve the energy,

$$P_{i}^{\mathrm{aux}}(t)=P_{a}^{\mathrm{aux}}(t)\sqrt{\max\left\{0,1-\frac{E_{ia}(t)}{T(t)}\right\}}$$ 58

Here $E_{ia}(t)=E_{i}(t)-E_{a}(t)$ is the inactive–active potential energy difference and *T*(*t*) is the kinetic energy. This definition of the inactive-potential momenta has, for instance, been used by Granucci et al. in their overlap-based decoherence approach.^36^

If the inactive-potential population falls below the threshold at time τ_d_ during the propagation, i.e., |*c*_*i*_(τ_d_)|^2^ < η, the wave packet is assumed to have fully decohered and its residual population is moved to the active potential by modifying the electronic coefficients as,

$$c_{a}(\tau_{d})=c_{a}(\tau_{d})\sqrt{1+\frac{|c_{i}(\tau_{d}){|}^{2}}{|c_{a}(\tau_{d}){|}^{2}}}$$ 59

$$c_{i}(\tau_{d})=0$$ 60

However, if the population of an inactive electronic potential rises above threshold at τ_b_ , |*c*_*i*_(τ_b_)|^2^ ≥ η, it is treated as the birth of a wave packet at *t* = τ_*b*_ . The auxiliary-momentum propagation is switched to the propagation according to eq 55, using the energy-rescaled active-potential auxiliary momentum from eq 58, *P*_*i*_^aux^(τ_*b*_), as initial condition. This allows for the interpretation that the newly born inactive-potential wave packet has been created by moving population from the active electronic potential while keeping the total energy along the trajectory constant.

This procedure facilitates the estimation of the FM and PFM decoherence rates along a trajectory initialized with a coherent superposition of several electronic states, passing through, possibly multiple, NAC regions, inducing surface hops. The interplay between these effects is approximately reflected as a series of births and deaths of inactive-potential wave packets that interact with the active-potential wave packet of the trajectory.

### 2.6 Momentum History Problem

In the outlined approach, at most a single wave packet is assigned to every inactive potential. It is represented by a single auxiliary momentum, the propagation of which depends on its entire history since the respective inactive potential population rose above the threshold η, eq 55. We expect this representation to work reasonably well for an inactive-potential population that has been created at time zero or by trespassing a NAC region, after which it monotonously decreases until it falls below the threshold η. This corresponds to departure of a single wave packet on the inactive potential until full decoherence, at which point the momentum propagation is reset to eq 58, losing the history.

Now consider the situation that the inactive-potential population, after a period of decoherence-induced decay, *t* = τ_*b*_ + Δτ_*d*_, receives a population increase, Δρ_*ii*_(*t*) = |*c*_*i*_(*t*)|^2^ – |*c*_*i*_(*t* – Δ*t*)|^2^ > 0, e.g., due to trespassing another NAC region. In the so far devised approach, the additional population, Δρ_*ii*_(*t*), will be assigned to the primary wave packet with the auxiliary momentum, *P* _*i*_^aux^(*t*), propagated from its birth at τ_*b*_ to *t* = τ_*b*_ + Δτ_*d*_ . At least for suitably large Δτ_*d*_ > 0, however, such an event should correspond to the birth of another wave packet with a newly initialized auxiliary momentum propagation rather than the amplification of the not yet fully decohered primary wave packet. To reflect this, we propose to modify the inactive-potential auxiliary momentum propagation according to eq 55 by injecting active-potential auxiliary momentum, rescaled for energy conservation, whenever the inactive-potential population increases. In this way, the auxiliary momentum for the added population, Δρ_*ii*_(*t*) = max {0, |*c*_*i*_(*t*)|^2^ – |*c*_*i*_(*t* – Δ*t*)|^2^}, is initialized as in eq 58,

$$P_{i}^{\mathrm{aux}}(t)=P_{i}^{\mathrm{aux}}(t)\left(1-\frac{\Delta \rho_{ii}(t)}{|c_{i}(t){|}^{2}}\right)+\frac{\Delta \rho_{ii}(t)}{|c_{i}(t){|}^{2}}\cdot P_{a}^{\mathrm{aux}}(t)\sqrt{\max\left\{0,1-\frac{E_{ia}(t)}{T(t)}\right\}}$$ 61

The so-obtained auxiliary momentum may be understood as an approximate average over the auxiliary momenta corresponding to all wave packets that are spawned at different times within τ_*b*_ → τ_*b*_ + Δτ_*d*_ but are propagated with the same auxiliary force, eq 55. A consequence of this description is that the injected momentum is set to zero if the potential energy gap exceeds the kinetic energy, effectively decelerating the overall auxiliary momentum. If, in turn, the inactive potential lies energetically below the active potential, the injected momentum is increased. To avoid further complications of the algorithm, we refrain from pursuing a finer treatment, which would require the propagation of multiple auxiliary momenta in every inactive potential, most likely in some similarity to a suggestion by Granucci et al.^36^ In the following, we use the notation TSH-FMi/PFMi to indicate that the active-potential auxiliary momentum injection is accounted for in fewest-switches TSH calculations with the FM/PFM decoherence rates, in contrast to TSH-FM/PFM where it is not.

### 2.7 Comparison with the TSH-EDC and TSH-ND Approaches

We compare TSH-FM/FMi and TSH-PFM/PFMi calculations against fewest-switches TSH calculations with the EDC proposed by Granucci and Persico^35^ as a simplification to the decoherence rate within the decay of mixing approach by Zhu, Truhlar and co-workers^32,33^ (TSH-EDC),

$$k_{ij}^{\mathrm{EDC}}=|E_{ij}|\cdot \frac{T}{T+C}$$ 62

It is determined by the absolute potential-energy difference, $|E_{ij}|,$ kinetic energy, *T*, and the parameter *C*. The EDC rate vanishes if the particles are at rest, *T* = 0, as do the FM and PFM decoherence rates, eqs 26 and 43. However, in the limit of high kinetic energies, *T* ≫ *C*, it approaches the potential-energy difference, *k*_*ij*_^EDC^ → $|E_{ij}|.$ The parameter *C* tunes the range of kinetic energies between the two limiting cases.

The EDC has been, at least implicitly, designed for situations in which the distribution of total energies in the initial conditions is rather narrow, for instance for processes excited with continuous-wave or narrow-band lasers, as well as molecular collisions.^35^ In particular, such laser-excitations usually only populate a single electronic potential. Electronic coherences thus arise only when the trajectories pass through a NAC region, e.g., an intersection seam at which the wave packet is split onto multiple electronic surfaces. Energy conservation keeps the total energy constant so that the wave packets after the NAC region will move with different kinetic energies, depending on the potential-energy difference with respect to the moment before the wave packet has been split. This is different from the situation encountered with an initial coherent superposition created by a broad-band laser, populating electronic potentials separated by several eV, up to a few tens of eV. Since the EDC rate is proportional to the potential-energy difference, its suitability for such cases with large potential-energy differences may be questioned from the formal perspective. However, the EDC has become a widely used decoherence correction for a manifold of TSH applications over the last decades, owing to the compromise between accuracy and computational simplicity that it provides.^5,15,16,19,20,46,47^ Hence, we employ the EDC as a TSH reference to test the FM and PFM decoherence rates, eqs 26 and 43, in particular regarding their ability to describe dynamics through conical intersections and in the long term.

To benchmark the survival of the initial coherence in the short-time dynamics, we also compare against conventional fewest-switches surface hopping without decoherence correction (TSH-ND). Upon-trajectory-averaging, this approach damps an initial coherence only by dephasing, representing an upper limit to the lifetime of an initial coherence that can be obtained from TSH simulations, at least as long as overcoherence artifacts can be neglected, e.g., at short times and in the absence of NAC close to the Franck–Condon region.

## 3 Overall Algorithm

The following algorithm has been implemented in a local modification of the SHARC package.^14,79^

1. Obtain an ensemble of *N*_IC_ initial conditions {***R***_ν_(0), ***P***_ν_(0)}, e.g., by sampling the Wigner distribution.^100^ In the 1D case, evaluate the variance, σ^2^, for the FM decoherence, eq 26. In the multidimensional case, evaluate ω, e.g. as the geometrical average over the normal-mode frequencies, eq 27, for the PFM decoherence, eq 43.
2. Loop over the initial conditions for which trajectories shall be propagated and for each one:
  a. Obtain the initial electronic coefficients *c*_*j*_(0).
  b. Identify the initial active potential, *a*(0), for each trajectory. We randomly collapse to one of the populated potentials, with probabilities |*c*_*j*_(0)|^2^.
  c. Evaluate the initial auxiliary momenta, *P* _*j*_^aux^(0), according to eqs 52 and 53 and set the initial auxiliary forces *F* _*j*_^aux^(0) = 0.
  d. Evaluate the initial set of adiabatic eigenstates, Ψ_*j*_(0), energies, $E_{j}(0),$ and the active-potential force, ***F***_*a*_(0).
  e. Loop over nuclear propagation time steps Δ*t*.
    i. Using ***F***_*a*_(*t*_0_), calculate midpoint velocities, $\overset{˙}{R}\left(t_{0}+\frac{\Delta t}{2}\right),$ and positions at the end of the time step, ***R*** (*t*_0_ + Δ*t*), with the first Velocity-Verlet half-step.
    ii. Evaluate Ψ_*j*_(*t*_0_ + Δ*t*), and $E_{j}$ ­(*t*_0_ + Δ*t*) at ***R*** (*t*_0_ + Δ*t*).
    iii. Evaluate auxiliary forces *F*_*j*_^aux^(*t*_0_) according to eq 51 using the midpoint velocity as the reference.
    iv. Following the local diabatization method,^91,101^ obtain the diabatization matrix for this time step, ***U*** (*t*_0_, *t*_0_ + Δ*t*). We use the approach implemented in the SHARC code,^79^ which constructs a unitary matrix ***U*** (*t*_0_, *t*_0_ + Δ*t*) such that for every state *j* the overlap ⟨Ψ̃_*j*_(*t*_0_ + Δ*t*)|Ψ_*j*_(*t*_0_)⟩ is maximized with a procedure detailed in the Supporting Information. The overall diabatization matrix is obtained as: *U*(0, *t*_0_ + Δ*t*) = *U*(*t*_0_, *t*_0_ + Δ*t*)*U*(0,*t*_0_).
    v. Obtain the NAC in the so-defined diabatic basis, ${\mathrm{K̃}}_{ij}(t_{0}+\Delta t)$ , according to eq 47 and transform the potential, *V*_*ij*_(*t*_0_ + Δ*t*) = δ_*ij*_ $E_{i}$ ­(*t*_0_ + Δ*t*), to the diabatic basis, *Ṽ*_*ij*_(*t*_0_ + Δ*t*).
    vi. Interpolate *Ṽ*_*ij*_, ${\mathrm{K̃}}_{ij}$ and ***U*** over the nuclear time step, [*t*_0_, *t*_0_ + Δ*t*], for which we use splines.
    vii. Store the active-potential index *a* ^′^ = *a*(*t*_0_) and enter the loop over electronic substeps δ*t*. We use 51 substeps in every nuclear time step.
      A. Propagate the electronic coefficients with the Runge–Kutta-4 method in the local diabatic basis at time *t* ^′^, *c*_*j*_(*t* ^′^) → *c̃*_*j*_(*t* ^′^ + δ*t*), using the interpolated diabatic potential matrix. Hereafter, the propagation is at the end of the electronic time step, *t* ^′^ + δ*t*. Drop time arguments for brevity.
      B. Using the interpolated ***U*** matrix, transform the electronic coefficients and the potential matrix to the adiabatic basis, *c*_*j*_ and *V*_*ij*_.
      C. Evaluate the hopping probability for every electronic potential, *P*_*a* → *i*_ . We use the density-flux formalism proposed by Petersen and Mitrić.^102^
      D. Draw a random number *r*. If ∑_*i* = 1_^*k*–1^ *P*_*a* → *i*_ < *r* ≤ ∑_*i* = 1_^*k*^ *P*_*a* → *i*_ conduct a surface hop, i.e., set *a* = *k*, when there is enough kinetic energy to cover the adiabatic potential-energy gap, *V*_*aa*_ + *T* ≥ *V*_*kk*_ . Else, the hop is frustrated, i.e., not carried out.
      E. Evaluate the FM, or PFM, decoherence rates according to eqs 26 and 43, or, alternatively, the EDC, eq 62, and apply the decoherence correction to the adiabatic electronic coefficients, eqs 48 and 49.
    viii. Calculate the active-potential force ***F***_*a*_(*t*_0_ + Δ*t*) at ***R*** (*t*_0_ + Δ*t*) and the velocities ***Ṙ*** (*t*_0_ + Δ*t*) with the second Velocity-Verlet half-step.
    ix. If the active potential has changed during the electronic propagation, *a* ^′^ ≠ *a*, rescale the velocity vector isotropically to conserve the total energy by setting
$$\overset{˙}{R}(t_{0}+\Delta t)=\overset{˙}{R}(t_{0}+\Delta t)\times \sqrt{1-\frac{\Delta E}{T(t_{0}+\Delta t)}}$$
      with the difference of total energies at the start and end of the time step defined as in eq 57.
    x. Propagate the active-potential auxiliary momentum, *P* _*a*_^aux^(*t*_0_ + Δ*t*), according to eqs 54 and 56.
    xi. Propagate inactive-potential auxiliary momenta, *P* _*i*_^aux^(*t*_0_ + Δ*t*), according to eq 55, if they carry above-threshold population, |*c*_*i*_(*t*_0_)|^2^ ≥ η and |*c*_*i*_(*t*_0_ + Δ*t*)|^2^ ≥ η, injecting active-potential momentum according to eq 61 if |*c*_*i*_(*t*_0_ + Δ*t*)|^2^ > |*c*_*i*_(*t*_0_)|^2^, when running TSH-FMi/PFMi simulations. If |*c*_*i*_(*t*_0_)|^2^ < η or |*c*_*i*_(*t*_0_ + Δ*t*)|^2^ < η, use eq 58.
    xii. If |*c*_*i*_(*t*_0_)|^2^ ≥ η and |*c*_*i*_(*t*_0_ + Δ*t*)|^2^ < η, the population fell below η during the time step. Move the remaining population to the active potential according to eqs 59 and 60.
3. After the propagation, identify valid trajectories by a suitable measure. We require energy conservation to be fulfilled within an energy-drift threshold, $E_{D}$ , over the whole propagation, Δ $E_{\mathrm{tot}}$ ­(*t*) = | $E_{\mathrm{tot}}$ ­(0) – $E_{\mathrm{tot}}$ ­(*t*) | ≤ $E_{D}$ , and exclude trajectories that violate this measure. If several electronic potentials are populated at time zero due to an initial coherent superposition, one has to take into account that trajectories started in different electronic potentials may be excluded with different probabilities. To mitigate this effect:
  a. Evaluate new fractions of active trajectories, eq 50, at time zero, Π_*j*_^valid^(0), after excluding all trajectories violating energy conservation.
  b. Identify the potential *k* with the highest probability of failure so that for all initially populated *j* ≠ *k*
$$\Pi_{k}(0)-\Pi_{k}^{\mathrm{valid}}(0)>\Pi_{j}(0)-\Pi_{j}^{\mathrm{valid}}(0)$$ 63
  c. Stochastically exclude trajectories initially started in potentials *j* ≠ *k* until the *t* = 0 fractions of active trajectories over the balanced ensemble of valid trajectories approximately match the original values,
$$\Pi_{j}^{\mathrm{valid},\mathrm{balanced}}(0)\approx \Pi_{j}(0)$$ 64
4. Evaluate observables by averaging over the so-obtained balanced ensemble of valid trajectories.

## 4 Computational Details

The resulting overall algorithm and details of the implementation^14,79^ are given in Section 3. We compare TSH-FMi/PFMi and TSH-FM/PFM simulations, respectively, with and without active-potential momentum injection, see Section 2.6, with TSH-ND (no decoherence correction) and TSH-EDC, proposed by Granucci and Persico.^35^ We employ the recommended parameter value *C* = 0.1 a.u. ≈ 2.72 eV^32^ in the EDC rate, eq 62, although there may be cases, in which a system-dependent choice is adequate.^94^ We consider zero temperature in all of our simulations.

For all molecules, we run all TSH simulation from a single set of ν = 1, ···, *N*_IC_ initial conditions {***R***_ν_(0), ***P***_ν_(0)}. However, we reinitialize the random-number generator for every trajectory, ensuring statistical independence of every trajectory for the stochastic selection of the initial active potential, *a*_ν_(0), and the surface-hopping procedure. With respect to these properties, the TSH trajectory ensembles obtained with different decoherence corrections are independent and we compare only their respective ensemble averages.

### 4.1 IBr

For the IBr molecule, we employ a diabatic model for the potential curves of the $1^{1}\Sigma_{0^{+}}^{+}$ ground and the 1Π0+3 and $1^{3}\Sigma_{0^{+}}^{-}$ excited states, as well as the NAC.^103^ Owing to the definition of the model, the quantum and TSH simulations have been carried out in the diabatic representation, however, all properties are analyzed in the adiabatic basis. The quantum-mechanical calculations have been conducted by solving the TDSE with the split-operator technique (splitting the kinetic and potential parts of the Hamiltonian) and the fast-Fourier-transform method for the evaluation of the kinetic part.^104^ We evaluated the numerical solution on a spatial grid with 1024 equidistant points between 3.5 a.u. and 11 a.u. using an integration time step of 0.01 fs, propagating until 200 fs.

The initial conditions for the quantum simulations have been obtained by promoting the diabatic $1^{1}\Sigma_{0^{+}}^{+}$ ground-state wave function, Ψ̃_0_(*r*), to the diabatic 1Π0+3 , Ψ̃_1_(*r*,0) = Ψ̃_0_(*r*), and $1^{3}\Sigma_{0^{+}}^{-}$ , Ψ̃_2_(*r*,0) = Ψ̃_0_(*r*) potentials, respectively. Quantum calculations have then been run for the three cases Ψ̃(*r*,0) = Ψ̃_1_(*r*,0), Ψ̃(*r*,0) = Ψ̃_2_(*r*,0), and $\mathrm{\Psi ̃}(r,0)=\frac{1}{\sqrt{2}}[{\mathrm{\Psi ̃}}_{1}(r,0)+{\mathrm{\Psi ̃}}_{2}(r,0)]$ . To mimick these initial conditions in the TSH simulations, we have sampled 10,000 initial positions and momenta from the exact numerical ground-state Wigner distribution of IBr, disregarding configurations with a Wigner function value lower than 10^–10^. From this ensemble we extracted a bond-length standard deviation of σ = 6.906 × 10^−2^ a.u., which we used to evaluate the FM decoherence rate. We ran the simulations by using the same diabatic-basis initial conditions as employed for the quantum simulations, using a nuclear propagation time step of 0.1 fs.

### 4.2 Polyatomic Molecules

In the TSH simulations of BMA­[5,5], para-xylene, fulvene, and glycine, we ran in each case trajectories from 1000 initial conditions obtained from the corresponding harmonic normal mode decomposition of each molecule. We employed the complete active space self-consistent field (CASSCF) electronic-structure method^105,106^ and analytic gradients implemented in the openMolcas package.^21^ The overlaps with the previous nuclear configuration, ⟨Ψ_*i*_(*t* + Δ*t*)|Ψ_*j*_(*t*)⟩ for the evaluation of the time-derivative coupling, eq 47, have been evaluated with the RASSI program^107^ within openMolcas under the assumption that the nuclear coordinates have not changed, i.e., updating only the molecular orbital and configuration interaction coefficients. To facilitate a direct comparison with the reference works of Vacher et al.^75^ (DD-vMCG calculations of BMA­[5,5] and para-xylene), and Ibele et al.^83^ (AIMS calculations for fulvene), we employed the same basis set, 6-31G*, and closely replicated the respective computational setups for the dynamics.

For BMA­[5,5] (C_10_H_12_), four π/π* orbitals localized on the terminal C–CH_2_ bonds have been included in the active space. The optimized geometry and harmonic normal-mode frequencies were obtained for the neutral ground state with the CASSCF­(4,4) approach. The geometric average over the 60 harmonic frequencies for the PFM decoherence correction has been obtained as ω_BMA_ = 5.463 × 10^–3^ a.u. (0.1487 eV, 1199.05 cm^–1^). We evaluated the first 30 fs of the dynamics after the initial coherent superposition of the lowest two adiabatic states of the BMA­[5,5] cation, $\Psi (0)=\frac{1}{\sqrt{2}}[\Psi_{1}(0)+\Psi_{2}(0)]$ , using a nuclear time step of 0.2 fs for all TSH approaches. In that, we incorporated the respective *D*_1_ and *D*_2_ cation states into a state-averaged CASSCF­(3,4) approach, abbreviated as SA(2)-CASSCF­(3,4), to evaluate the electronic structure.

For para-xylene (C_8_H_10_), the same setup is used for the TSH dynamics, albeit including six π/π* orbitals into the active space, i.e., using SA(2)-CASSCF­(5,6) electronic structure calculations.^75^ The optimized geometry and harmonic normal-mode frequencies have been evaluated for the neutral ground state with the second order many-body perturbation theory (MBPT2) method implemented in openMolcas,^21^ since two negative frequencies were obtained with the CASSCF­(6,6) approach. The geometric average over the 48 normal-mode frequencies has been obtained as ω_pXyl_ = 4.766 × 10^–3^ a.u. (0.1297 eV, 1045.91 cm^–1^).

For fulvene (C_6_H_6_) we incorporated again six π/π* orbitals into the active space, according to the reference.^83^ The optimized ground-state geometry and harmonic normal-mode frequencies were obtained with the CASSCF­(6,6) electronic structure method. The geometric average over the 30 frequencies has been obtained as ω_ful_ = 5.467 × 10^–3^ a.u. (0.1488 eV, 1199.93 cm^–1^). We aim to study the stepwise back-transfer of an initial *S*_1_ wave packet through the sloped conical *S*_1_/*S*_0_ intersection, due to which fulvene has been identified as a molecular representation of Tully’s model system III.^83^ To this end, we evaluate the first 50 fs of the dynamics after the population has been initialized in the *S*_1_ state with SA(2)-CASSCF­(6,6) electronic structure calculations. We use a nuclear time step of 0.1 fs, which has been identified as sufficient to avoid stepping over the narrow intersection.^47^ To favor the transfer through the sloped topology of the *S*_1_/*S*_0_ intersection seam, the initial momenta of all initial conditions have been set to zero.^83^

For glycine (C_2_H_5_NO_2_), we employ the cc-pVDZ basis set. The optimized geometry and harmonic normal-mode frequencies for the ground state of the I_*p*_ conformer of glycine^108^ have been obtained with the MBPT2 method in the openMolcas package. The geometric average over the 24 normal-mode frequencies has been obtained as ω_gly_ = 4.814 × 10^–3^ a.u. (0.1310 eV, 1056.51 cm^–1^). For the TSH simulations we employed SA(5)-CASSCF­(6,4) electronic structure calculations, including two π/π* and two σ orbitals into the active space. Using a time step of 0.3 fs we evaluated the first 100 fs of the dynamics following an initial coherent superposition of the *S*_1_ and *S*_2_ states, $\Psi (0)=\frac{1}{\sqrt{2}}[\Psi_{1}(0)+\Psi_{2}(0)]$ .

The optimized geometries and frequencies for all molecules are given in the Supporting Information. The distributions of total energies obtained for all molecules at *t* = 0 from the respective ensembles of initial conditions are given in Appendix [Sec secA.1]. For IBr, the quantum distributions of total energies are given there as well.

### 4.3 Energy Conservation

For the determination of the balanced valid trajectories as described in Section 3, we require that the total energy is conserved within $\Delta E_{tot}(t)=|E_{tot}(0)-E_{tot}(t)|$ ≤ 1.0 eV over the whole trajectory. Trajectories that violate this threshold are excluded from the analysis. As detailed in the Supporting Information, no trajectories are excluded in the TSH-FMi/PFMi calculations for IBr, BMA­[5,5] and para-xylene, even when using a much smaller threshold of 0.1 eV, while for fulvene, only about 2.5% of the trajectories are excluded with Δ E _tot_(*t*) ≤ 1.0 eV and 6.0% with ΔE _tot_(*t*) ≤ 0.1 eV. For these molecules, we obtain conservation of the ensemble-averaged total energy within ± 0.002 eV of the initial value.

For glycine, Δ E _tot_(*t*) ≤ 1.0 eV leads to an exclusion of about 20% of the trajectories. To retain large enough ensembles we thus have selected 1.0 eV as the threshold for all molecules for the sake of simplicity. The ensemble-averaged total energy obtained with this threshold for glycine is within ±0.01 eV of the original value for the first 15 fs of the propagation. As we will show below, this is the time region relevant to describe the evolution of the initial electronic coherence for all investigated molecules, which is the main focus of the present work. The conservation of energy becomes progressively worse at longer times for glycine. The deviation from the initial total energy increases to between 0.1 and 0.2 eV after 100 fs, depending on the selection of initial active potentials and decoherence correction. This is likely due to instabilities in the active space, which manifest at longer propagation times. However, this will not affect the discussion that follows.

## 5 Results and Discussion

### 5.1 Numerical Consistency and Convergence

We first investigate the convergence of the FM and PFM decoherence rates with respect to the inactive-potential population threshold parameter, η, introduced in Section 2.5 and the effect of the active-potential momentum injection, introduced in Section 2.6. If the inactive-potential population is larger than η, it is assumed to harbor a wave packet, the auxiliary momentum of which is propagated according to the auxiliary inactive-potential forces. We first investigate the one-dimensional IBr molecule with the FM decoherence, since all inactive-potential forces can be evaluated numerically exact. In this way, we hope to avoid interferences with further approximations made in the derivation of the PFM decoherence onto the convergence study. The model is illustrated in Figure 1a. It consists of the three lowest Ω = 0^+^ adiabatic potentials obtained from a diabatic model.^103^ We consider the scenario in which the nuclear wave packet is instantaneously promoted from the ${1\;}^{1}\Sigma_{0^{+}}^{+}$ ground state to the 1Π0+3 excited state and goes through a spin–orbit coupling (SOC)-induced avoided crossing with the ${1\;}^{3}\Sigma_{0^{+}}^{-}$ state. At the avoided crossing, the nuclear wave packet splits as a consequence of which two dissociation channels are accessible, I+Br, and I+Br*, the latter leaving the Br atom in an excited state.

**Figure 1:**
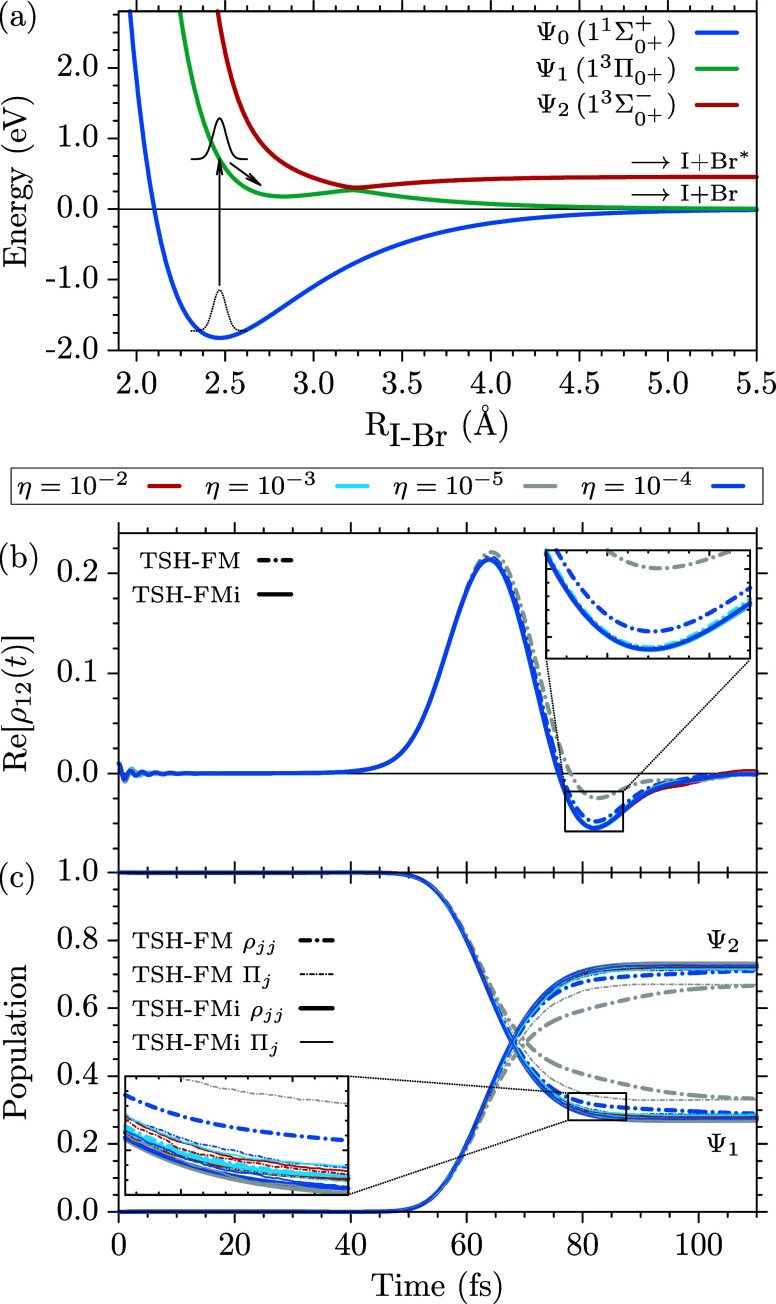
(a) Adiabatic potential energy curves of the three lowest Ω = 0^+^ electronic states of IBr obtained from the diabatic model of ref 103. The wave packet is instantaneously promoted from the ${1\;}^{1}\Sigma_{0^{+}}^{+}$ ground state (Ψ_0_, blue) to the 1Π0+3 excited state (Ψ_1_, turquoise). It goes through a spin–orbit-induced avoided crossing with the ${1\;}^{3}\Sigma_{0^{+}}^{-}$ state (Ψ_2_, red), due to which two final dissociation channels are accessible, I+Br, and I+Br*, see text. (b) Coherence between adiabatic electronic states Ψ_1_ and Ψ_2_, represented by the real part of the off-diagonal density matrix element Re­[ρ_12_(*t*)]. (c) Averaged Ψ_1_ and Ψ_2_ adiabatic populations ρ_*jj*_ (thick) and fractions of active trajectories Π_*j*_ (thin), see eq 50, using the same style and colors as in (b). All results were averaged over 10^4^ independent trajectories and the dynamics was initialized in the diabatic state Ψ̃(0) = Ψ̃_1_(0). Panels (b) and (c) show a comparison of the nonadiabatic dynamics predicted by TSH-FM (dash-dotted lines) and TSH-FMi (solid lines) for inactive-potential population thresholds η = 10^–2^, 10^–3^, 10^–4^, 10^–5^, indicated by the colors shown in the legend.

To investigate the impact of the inactive-population threshold parameter, η, on the TSH simulations of this dynamics with (TSH-FMi) and without (TSH-FM) active-potential momentum injection, we calculated for both cases independent sets of 10^4^ trajectories for η = 10^–2^, 10^–3^, 10^–4^, 10^–5^, started in the diabatic potential of the 1Π0+3 state. The coherence between the adiabatic 1Π0+3 and ${1\;}^{3}\Sigma_{0^{+}}^{-}$ excited states, given by the real part of the off-diagonal density matrix element Re­[ρ_12_(*t*)], is shown in Figure 1b. It develops as the nuclear wave packet goes through the conical intersection and the adiabatic population of the ${1\;}^{3}\Sigma_{0^{+}}^{-}$ state, Figure 1c, starts to rise. In the case of the TSH-FMi simulations, including the active-potential momentum injection into the propagation of the inactive-potential momenta, the results for different values of η ≤ 10^–3^ are undistinguishable, with very minute differences for η = 10^–2^ for *t* > 90 fs. More substantial differences are observed in TSH-FM calculations, without active-potential momentum injection. Here, one needs to employ larger inactive-potential population thresholds of η = 10^–2^ or 10^–3^, with which the TSH-FM results are undistinguishable from the TSH-FMi ones. η = 10^–4^ leads to small differences, while η = 10^–5^ leads to a considerable deviation from the TSH-FMi results. Note that the small oscillations visible in the first 8 fs in Figure 1b stem from the initialization in the diabatic basis representation of the state 1Π0+3 , Ψ̃_1_(0). The reason is that the NAC in the IBr model is constant,^103^ so that transformation to the adiabatic basis produces tiny but nonzero occupations of the other adiabatic state, ${1\;}^{3}\Sigma_{0^{+}}^{-}$ , even at *t* = 0.

To elucidate the internal consistency of the simulation, see Section 2.4, Figure 1c shows the comparison between the averaged adiabatic populations, ρ _*jj*_, and the fractions of active trajectories, Π_*j*_ , defined in eq 50. It shows that the TSH-FMi simulations, with active-potential momentum injection, are internally consistent for all inactive-potential population threshold parameters. However, the TSH-FM simulations are internally consistent only for η = 10^–2^ and 10^–3^.

These results demonstrate that the active-potential momentum injection can indeed alleviate the momentum history problem of the inactive-potential momentum propagation, as discussed in Section 2.6, due to which the TSH-FMi calculations are rather insensitive to the inactive-potential population threshold η. Due to this, we use active-potential momentum injection (FMi/PFMi) for all further studies in this work in combination with a small inactive-potential population cutoff parameter η = 10^–4^.

To verify the active-potential momentum-injection for use in larger systems in the case of an initial coherent superposition of electronic states, we have studied the dynamics induced in the I_*p*_ conformer of glycine^108^ by a symmetric and even superposition of the *S*_1_ and *S*_2_ excited states, $\Psi \left(0\right)=\frac{1}{\sqrt{2}}\left[\Psi_{1}\left(0\right)+\Psi_{2}\left(0\right)\right]$ . Employing the inactive-potential momentum propagation threshold η = 10^–4^, determined for the case of IBr, we simulated the first 100 fs of the dynamics by calculating 1000 trajectories, see Section 4 for details, with the TSH-PFMi and TSH-PFM methods, i.e., with and without active-potential momentum injection, respectively. After removing those trajectories that violate energy conservation by more than 1.0 eV and balancing the remainder, see Section 3, we obtain 797 valid trajectories for TSH-PFM and 816 for TSH-PFMi. Figure 2a shows the *S*_1_–*S*_2_ electronic coherence as the real part of the ensemble-averages over the off-diagonal density matrix element Re­[ρ_12_(*t*)] obtained with the TSH-PFMi and TSH-PFM approaches. Due to the even distribution of the *S*_1_ and *S*_2_ populations at *t* = 0, the initial coherence is 0.5. The value of the coherence rapidly oscillates during the first few femtoseconds, with a frequency that roughly corresponds to the energy difference between the *S*_1_ and *S*_2_ states. Dephasing, due to the trajectory average, and the PFM decoherence correction, approximating the decay of nuclear overlap, damp the oscillations into the confidence interval of the ensemble average within about 5 fs. The differences between the coherences predicted by the TSH-PFM and TSH-PFMi method are only visible in the blow up in Figure 2a. Figure 2b depicts the corresponding averaged adiabatic populations, ρ_11_ and ρ_22_, and fractions of active trajectories, Π_1_ and Π_2_. The average populations of the *S*_1_ and *S*_2_ states remain close to 0.5 in the plotted time interval, although a small *S*_2_ → *S*_1_ population transfer of about 5% (PFM) and 7% (PFMi) is observed. Further, the *S*_1_ and *S*_2_ populations obtained with the TSH-PFMi approach are larger/smaller by about 0.02 than those obtained with TSH-PFM. At least partially, these small differences can be due to the independent stochastic selection of the initial active potential in both sets of trajectories, leading to a small deviation of the respective fractions of active trajectories at the beginning, which is kept through later times. The adaptation of the adiabatic populations to the fractions of trajectories within the first about 4 fs of the dynamics is due to the fact that the stochastic selection of initial active potentials does not produce an exact 50:50 distribution of active trajectories. Internal consistency is obtained with both approaches.

**Figure 2:**
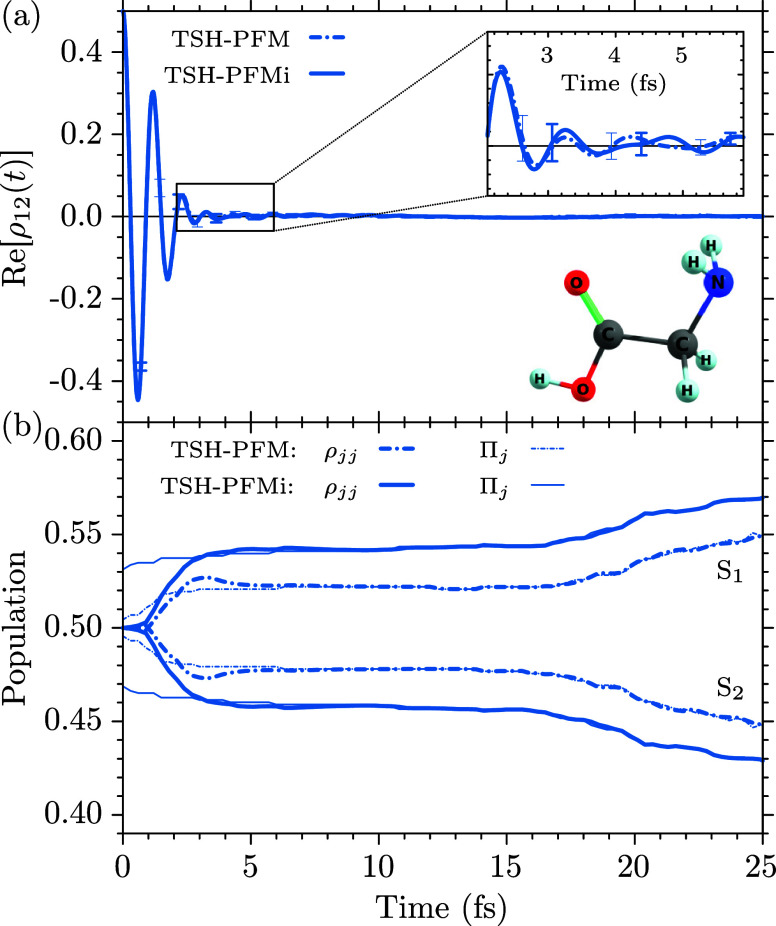
Nonadiabatic dynamics in the I_*p*_ conformer of glycine induced by the symmetric superposition of the *S*_1_ and *S*_2_ excited states, $\Psi \left(0\right)=\frac{1}{\sqrt{2}}[\Psi_{1}\left(0\right)+\Psi_{2}\left(0\right)]$ predicted by TSH-PFM (dash-dotted lines) and TSH-PFMi (solid lines). (a) *S*_1_–*S*_2_ coherence, represented by the real part of the off-diagonal density matrix element Re­[ρ_12_(*t*)]. (b) Average adiabatic populations ρ_*jj*_ (thick) and fractions of active trajectories Π_*j*_ (thin), see eq 50. The TSH-PFM and TSH-PFMi results have been averaged over 797 and 816 trajectories, respectively. An inactive-potential population threshold η = 10^–4^ has been employed. The error bars in panel (a) depict the 95% confidence intervals of the averages.

These results indicate that for η = 10^–4^, the TSH-PFM and TSH-PFMi simulations predict very similar dynamics in glycine. Taking this into consideration together with the fact that a considerably weaker dependence on the inactive-potential population threshold, η, has been observed for IBr when including the active-potential momentum injection (see above), we choose to use the inactive-potential momentum propagation threshold η = 10^–4^ with the TSH-FMi/PFMi approaches in what follows.

### 5.2 Tests of the Model

In this subsection, we first benchmark the TSH-FMi method for the linear molecule IBr to scrutinize the approximations made in the derivation of the FM decoherence rate, eq 26. In that, we compare against fully quantum-mechanical calculations for the passage through a conical intersection of an initial electronic coherence as well as of wave packets initialized in a single electronic excited state. In the second part of the section, we test the additional approximations in the derivation of the PFM decoherence rate for multidimensional systems, eq 43. To this end we compare TSH-PFMi simulations of the nonadiabatic dynamics of initial electronic coherences in the polyatomic molecules BMA­[5,5] and para-xylene against DD-vMCG calculations by Vacher et al.^75^ Further, TSH-PFMi simulations of the *S*_1_-excited state population dynamics in fulvene, a molecular Tully-III model characterized by (repeated) passage through a steep *S*_0_–*S*_1_ conical intersection followed by reflection into the crossing region, are compared against AIMS reference calculations reported by Ibele et al.^83^ We also compare in all cases against results obtained with fewest-switches TSH without decoherence correction (TSH-ND)^1,2^ and using TSH-EDC,^35^ representing conventional TSH methods designed for situations with a narrow distribution of total energies in the ensemble. Their application to simulate the dynamics after an initial coherent electronic superposition spanning a broad range of total energies presents a limiting case which we adopt in order to put the performance of the TSH-PFM approach, which has been designed for broad distributions of total energies, into perspective. This comparison is extended to the glycine molecule, for which we additionally investigate how the rapid coherent electron dynamics induced by the initial coherence manifests in the molecular dipole, i.e., an observable. The initial total-energy distributions for all studied systems are given in Appendix [Sec secA.1].

#### 5.2.1 IBr

Using the same diabatic-basis model^103^ as in Section 5.1, depicted in Figure 1a, we simulated the nonadiabatic dynamics in IBr with the TSH-FMi, TSH-ND, and TSH-EDC methods, and performed full-quantum simulations following (i) a symmetric coherent superposition of the diabatic $1^{3}\Pi_{0^{+}}$ (Ψ̃_1_) and 1Σ30+− (Ψ̃_2_) states, $\mathrm{\Psi ̃}\left(0\right)=\frac{1}{\sqrt{2}}\left[{\mathrm{\Psi ̃}}_{1}\left(0\right)+{\mathrm{\Psi ̃}}_{2}\left(0\right)\right]$ , (ii) full population of the ${1\;}^{3}\Pi_{0^{+}}$ state, Ψ̃(0) = Ψ̃_1_(0), and (iii) of the 1Σ30+− state, Ψ̃(0) = Ψ̃_2_(0). For the TSH simulations we ran 10^4^ trajectories and employed the inactive-potential momentum propagation threshold η = 10^–4^ for the TSH-FMi simulations.

Panels (a), (b), and (c) of Figure 3 show for all methods the evolution of the coherence between the adiabatic states, 1Π30+ and $1^{3}\Sigma_{0^{+}}^{-}$ , represented by the real part of the density-matrix element, Re­[ρ_12_(*t*)], when starting, respectively, from the coherent superposition, the 1Π30+ potential, and the ${1\;}^{3}\Sigma_{0^{+}}^{-}$ potential, all defined in the diabatic basis. Panels (d), (e), and (f) of Figure 3 depict the corresponding adiabatic populations, ρ_*jj*_ , and fractions of active trajectories, Π_*j*_ , in the same order. For the TSH simulations, all data correspond to ensemble averages over 10^4^ trajectories. The results of the full quantum mechanical calculation in Figure 3a show that the coherence, starting at its maximum value of 0.5, rapidly oscillates, while its magnitude is damped to almost zero within 8 fs.

**Figure 3:**
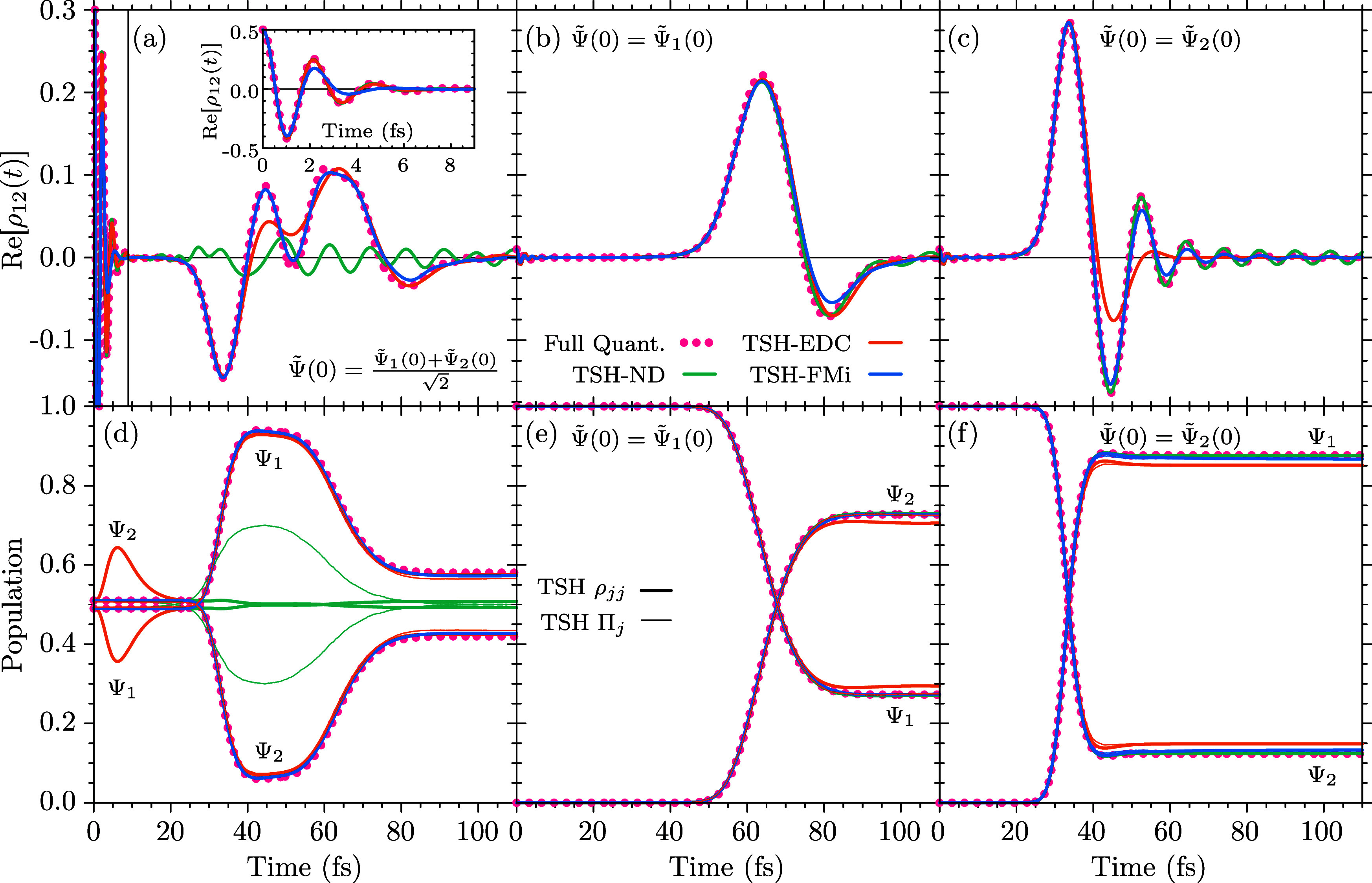
Nonadiabatic dynamics in IBr using the same potential energy curves and couplings as in Figure 1a. The top row, panels (a–c), depict the evolution of the coherence between the adiabatic electronic states Ψ_1_ and Ψ_2_ , i.e., the real part of the density-matrix element, Re­[ρ_12_(*t*)], resulting from full quantum mechanical (red, dotted), TSH-ND (turquoise), TSH-EDC (orange), and TSH-FMi (blue) calculations for (a) an initial symmetric superposition of the diabatic-basis excited states, $\mathrm{\Psi ̃}(0)=\frac{1}{\sqrt{2}}[{\mathrm{\Psi ̃}}_{1}(0)+{\mathrm{\Psi ̃}}_{2}(0)]$ , (b) initial population of the first diabatic excited state, Ψ̃(0) = Ψ̃_1_(0), and (c) for Ψ̃(0) = Ψ̃_2_(0). The bottom row, panels (d–f) show, using the same color code, the averaged adiabatic populations ρ_*jj*_ (thick lines) and the fraction of active trajectories Π_*j*_ (thin lines), see eq 50, of the adiabatic states Ψ_1_ and Ψ_2_ for the same initial conditions used in panels (a–c). All TSH results were obtained by averaging over 10^4^ independent trajectories. An inactive-potential population threshold of η = 10^–4^ was employed for TSH-FMi.

Within this time, the first full oscillation period takes about 2.1 fs, the second about 2.5 fs, and the third about 3 fs, reflecting the fact that the energy difference between the potential surfaces, see Figure 1a, is reduced as the conical intersection is approached. The damping of the coherence occurs because of the different slopes of the potential surfaces, due to which the wave packets propagating on either surface loose their overlap within about 8 fs and arrive at the conical intersection at different times. As can be deduced together with the populations drawn in Figure 3d, the faster wave packet traveling on the ${1\;}^{3}\Sigma_{0^{+}}^{-}$ potential arrives after about 25 fs at the conical intersection, leading to the onset of the coherent trace and an almost full ${1\;}^{3}\Sigma_{0^{+}}^{-}\to {1\;}^{3}\Pi_{0^{+}}$ population transfer reaching its maximum at about 40 fs. After a plateau region in the populations until about 50 fs, the slower wave packet, traveling in the 1Π0+3 potential, arrives at the conical intersection, transferring the majority of its population to the ${1\;}^{3}\Sigma_{0^{+}}^{-}$ potential. This induces the second part of the coherent trace of the passage through the conical intersection that lasts until about 100 fs. Comparison with the coherent traces and population dynamics when starting the propagation in the individual states ${1\;}^{3}\Pi_{0^{+}}$ , Figure 3b,e, and ${1\;}^{3}\Sigma_{0^{+}}^{-}$ , Figure 3c,f, confirms that the coherence and population dynamics of the wave packet’s passage through the conical intersection can be approximately represented by a linear combination of the individual-state results. The reason for this is that the wave packet has fully decohered before reaching the conical intersection.

The quantum result for the decay of the initial coherence during the first 8 fs is reproduced perfectly with the TSH-EDC and TSH-ND approaches, while the TSH-FMi calculation shows a slightly faster decoherence. This may be related to the fact that the positions of the auxiliary wave packets on the inactive potentials have been chosen such as to maximize the decoherence in the derivation of the FM decoherence formula, see the original work,^34^ and the discussion around eq 24. Interestingly, the TSH-EDC adiabatic populations show significant dynamics in the first 20 fs, before the conical intersection is reached, with a transient ${1\;}^{3}\Pi_{0^{+}}\to {1\;}^{3}\Sigma_{0^{+}}^{-}$ transfer of about 15% of the population. This temporarily breaks internal consistency for TSH-EDC, while the other methods predict internally consistent adiabatic populations and fractions of active trajectories, ρ_*jj*_ = Π_*j*_ , see eq 50, of 0.5 in this part of the propagation. Due to decoherence slowly taking over, the TSH-EDC adiabatic populations eventually return to 0.5 after 20 fs, since the respective hops to the higher potential surface are frustrated, i.e., not carried out.

The quantum results for the emerging coherence and population dynamics during the wave packet’s passage through the conical intersection are reproduced almost perfectly with the TSH-FMi approach, while TSH-EDC underestimates the buildup of the coherence between 40 and 60 fs. As can be seen in Figure 3c, this is due to a too fast decay of the coherence built up by the passage through the conical intersection of the wave packet initialized on the steeper ${1\;}^{3}\Sigma_{0^{+}}^{-}$ potential, possibly because its higher kinetic energy leads to a larger EDC decoherence rate than for the wave packet initialized in the ${1\;}^{3}\Pi_{0^{+}}$ potential. The TSH-ND approach fails to describe this region of the dynamics, since the initial coherence is not removed from the individual trajectories before arriving at the conical intersection, leading to overcoherence artifacts and an internally inconsistent propagation.

When the dynamics starts from the ${1\;}^{3}\Pi_{0^{+}}$ state, all three TSH methods provide a similar good description of the coherences and the (internally consistent) adiabatic populations in the whole time range. The same applies for the dynamics starting from the ${1\;}^{3}\Sigma_{0^{+}}^{-}$ state, apart from the aforementioned overestimation of the decoherence by the TSH-EDC approach. The TSH-ND results underline the need to take into account decoherence corrections into TSH simulations, which has been the reason for the decade-long developments leading among others to the EDC.^23–44^ Across the three cases of nonadiabatic dynamics studied in IBr, the TSH-FMi results are overall closest to the quantum reference, imperfected only by the slight overestimation of the decoherence of the initial electronic wave packet.

#### 5.2.2 BMA­[5,5] and para-Xylene Cations

BMA­[5,5] (C_10_H_12_) is a modified bismethyl-adamantane (BMA) molecule, with its cage consisting of two interconnected five-member carbon rings; see scheme in Figure 4a. Para-xylene (CH_3_–C_6_H_4_–CH_3_) is composed of a phenyl ring and two methyl groups in para position, see scheme in Figure 5a. For both molecules, Vacher et al.^75^ employed the DD-vMCG method to calculate the coupled electron nuclear dynamics of an initial symmetric superposition of the lowest two electronic states of their respective cations, *D*_1_ and *D*_2_, arising from ionization of π molecular orbitals. In this method, both, the electronic and nuclear degrees of freedom, are treated quantum mechanically by propagating coupled frozen Gaussian wave packets on the electronic potential surfaces, which are computed on the fly.^77,78^ Due to this, the results reported in ref 75 likely represent the best available approximation to a full quantum-mechanical treatment for these molecules, and we employ them as our reference.

For a meaningful comparison with the DD-vMCG results^75^ we employ the same, respective, CASSCF electronic structure for both molecules, see computational details in Section 4.2. For both molecular cations, we evaluated the first 30 fs of the dynamics after the initial coherent superposition, $\Psi (0)=\frac{1}{\sqrt{2}}[\Psi_{1}(0)+\Psi_{2}(0)]$ , using 1000 trajectories for the TSH-ND, TSH-EDC and the TSH-PFMi approach with the inactive-potential momentum propagation threshold η = 10^–4^. All trajectories have been found to conserve energy within 1.0 eV, and, consequently have been used for the ensemble averages.

Figures 4a and 5a show for the BMA­[5,5] and, respectively, para-xylene cations the time evolution of the *D*_1_-*D*_2_-coherence represented by twice the real part of the off-diagonal electronic density matrix element, 2Re­[ρ̃_12_(*t*)], in the diabatic basis, to be consistent with the reference DD-vMCG data.^75^ The respective panels (b) depict the adiabatic *D*_2_ populations, ρ_22_ , and fraction of active trajectories, Π_2_. As in the reference data,^75^ the coherences start with a value of 1.0 and rapidly oscillate in time with decreasing amplitude.

For the BMA­[5,5] cation, the TSH-PFMi and TSH-ND methods provide almost perfect agreement to the DD-vMCG reference for the first 3.5 fs, after which they exhibit a stronger damping of the initial coherence than the DD-vMCG reference. This is most likely because the DD-vMCG calculations account for the quantum nature of the nuclei beyond a simple estimation of the nuclear overlap decay, which may even slow down the decay of the initial coherence.^75^ However, one should also take into account that the DD-vMCG method might, depending on the simulation parameters, also overestimate coherences to some degree, which is difficult to assess without a true full-quantum calculation for these systems.

**Figure 4:**
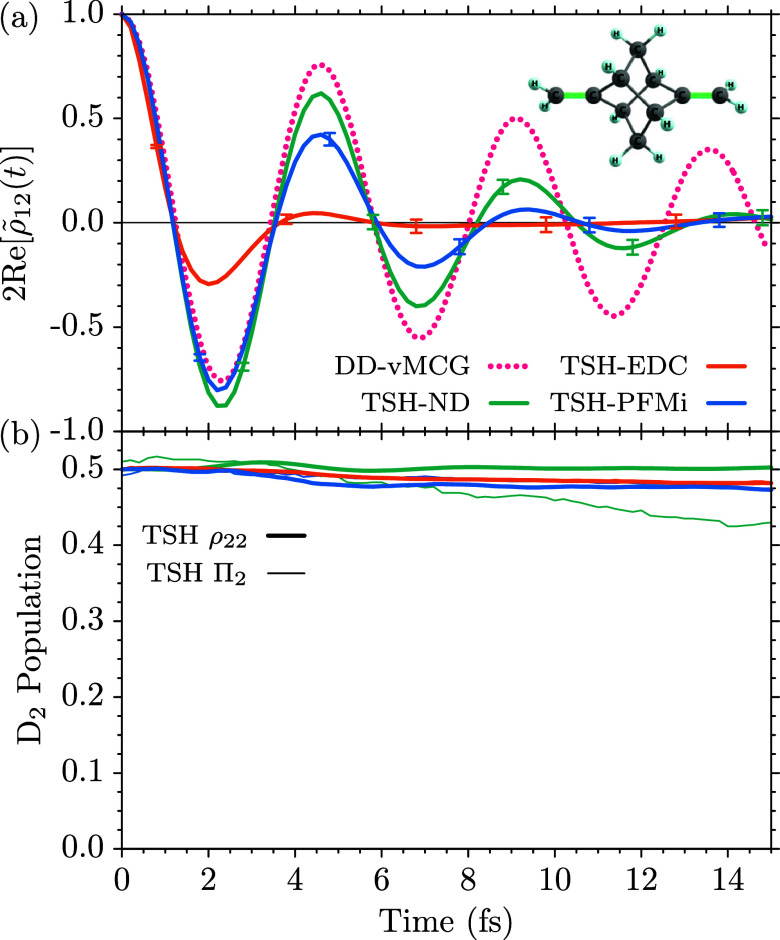
Coupled electron–nuclear dynamics induced by a symmetric superposition of the two lowest states, *D*_1_ and *D*_2_ , of the BMA­[5,5] cation, $\Psi (0)=\frac{1}{\sqrt{2}}[\Psi_{1}(0)+\Psi_{2}(0)]$ . (a) Evolution of the electronic coherence in the diabatic basis, represented by twice the real part of the off-diagonal electronic density matrix element, 2Re­[ρ̃_12_(*t*)], resulting from a DD-vMCG simulation (red dotted), digitized from Vacher et al.,^75^ as well as TSH-ND (turquoise), TSH-EDC (orange), and TSH-PFMi (blue) calculations. For the latter, an inactive-potential population threshold of η = 10^–4^ was used. (b) Averaged adiabatic TSH populations, ρ_22_ , (thick) and fraction of active trajectories, Π_2_, (thin), see eq 50, for the *D*_2_ state. All TSH results were obtained by averaging over 10^3^ trajectories. Error bars indicate the 95% confidence intervals of the TSH averages.

**Figure 5:**
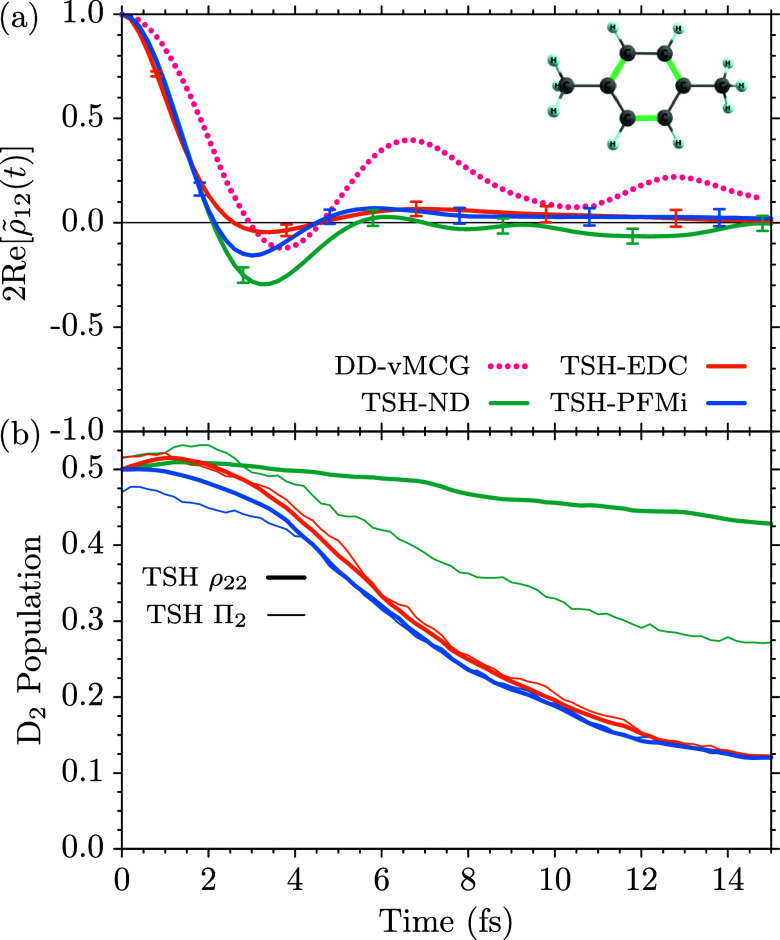
Coupled electron–nuclear dynamics induced by a symmetric superposition of the two lowest states, *D*_1_ and *D*_2_, of the para-xylene cation, $\Psi (0)=\frac{1}{\sqrt{2}}[\Psi_{1}(0)+\Psi_{2}(0)]$ . (a) Evolution of the electronic coherence, represented by twice the real part of the off-diagonal electronic density matrix element, 2Re­[ρ̃_12_(*t*)], resulting from a DD-vMCG simulation (red, dotted) digitized from Vacher et al.,^75^ TSH-ND (turquoise), TSH-EDC (orange), and TSH-PFMi (blue) using an inactive-potential population threshold of η = 10^–4^. (b) Averaged adiabatic population ρ_22_ (thick) and fraction of active trajectories Π_2_ (thin), see eq 50, for the *D*_2_ state. All TSH results were obtained by averaging over 10^3^ trajectories. Error bars indicate the 95% confidence intervals of the TSH averages.

The TSH-PFMi results exhibit stronger decoherence than the TSH-ND result without decoherence correction, which is somewhat expected since the latter represents the maximum amount of coherence that can be obtained from TSH simulations. Still, both approaches predict visible coherent oscillations until 14 fs of the dynamics. The TSH-EDC results, show a much faster damping of the initial coherence, reaching less than 50% of the TSH-ND amplitude in the first oscillation, and less than 10% after about 5 fs, before the oscillation is completely damped after 6 fs.

For the para-xylene cation, none of the TSH methods provides good agreement with DD-vMCG^75^ regarding the evolution of the initial coherence beyond 6 fs, as all predict a too-fast damping. At earlier times, the TSH-PFMi approach provides the best overall agreement with the DD-vMCG reference, representing an intermediate between the TSH-EDC and the TSH-ND results which, respectively, predict less and more pronounced first coherent oscillations than in the reference data.

The much faster damping of the coherent oscillation in para-xylene reflects the characteristics of the involved electronic potentials. The *D*_1_ and *D*_2_ states of the BMA­[5,5] cation have similar slopes in the Franck–Condon region, while the opposite is true for the para-xylene cation, whose *D*_1_ and *D*_2_ potential surfaces are inclined with respect to each other.^74,75^ For the BMA­[5,5] cation, one thus expects a weak electronic decoherence leading to a long survival of the initial coherence. For para-xylene, however, a fast decoherence is expected. The adiabatic *D*_2_ populations obtained with the TSH approaches reflect these different characteristics to some degree. They remain close to 0.5 for the BMA­[5,5] cation for the depicted 15 fs of the dynamics. Here the TSH-PFMi and TSH-EDC results are internally consistent, while the TSH-ND simulation predicts a slowly increasing number of surface-hops to the *D*_1_ state, leading to a small but increasing internal inconsistency. For the para-xylene cation, the adiabatic TSH-EDC and TSH-PFMi populations drop to about 0.12 after 15 fs of the dynamics, maintaining internal consistency for the whole time, while the TSH-ND populations show a much slower population transfer and considerable internal inconsistency. This indicates that the NAC between the *D*_1_ and *D*_2_ states of the BMA­[5,5] cation is almost negligible, while it is rather pronounced for the para-xylene cation. In the latter case, the overly coherent character of the TSH-ND simulation thus becomes apparent in its broken internal consistency.

The results for these two molecules show that the TSH-PFMi approach predicts the evolution of initial coherences in reasonable agreement with DD-vMCG reference calculations given the inherent limitations by the classical treatment of the nuclei in TSH. Albeit the TSH-ND method can provide a slightly longer survival of an initial coherence, it can not maintain internal consistency, making it unsuitable for the evaluation of coherence-sensitive observables, see Section 2.4. TSH-PFMi seems to predict only a slightly shorter survival of initial coherences than the TSH-ND method, while it coincides at longer times, after the decay of the initial coherence, with TSH-EDC. Further, the population dynamics obtained by the TSH-PFMi and TSH-EDC methods are essentially the same and, in particular, internally consistent, allowing for the evaluation of observables based on the electronic coefficients/populations, see Section 2.4.

#### 5.2.3 Fulvene

The fulvene molecule, CH_2_–C_5_H_4_, is a cross-conjugated hydrocarbon constituted by a pentagonal ring and a methylidene sharing an exocyclic double bond. Its lowest excited state, *S*_1_, yields a conical intersection seam into the ground state, *S*_0_ , through which fast internal conversion occurs via two mechanisms, an elongation of the C–C bond of the CH_2_ moiety, or a torsional motion.^109,110^ The bond elongation leads to a region of the intersection seam with a strongly sloped topology, while the torsional motion leads to a region of the seam with a peaked topology, with distinct characteristics.^111^ The passage through the sloped CI has been identified as the more probable of both mechanisms.^111^ Owing to its topology, the portion of the wave packet transferred to *S*_0_ is reflected back into the intersection seam, leading to a stepwise *S*_1_ → *S*_0_ population transfer that is reminiscent of Tully’s model III.^2,83^ This back and forth motion of the nuclear wave packet through the conical intersection poses a challenge to its theoretical description, due to which it has been used to benchmark the DD-vMCG, AIMS, and various TSH methods.^47,83,111,112^

This system shall serve as a test of the TSH-PFMi approach regarding its ability to predict such complex dynamics without an initial coherent superposition in comparison with a high-level AIMS calculation, reported by Ibele et al.^83^ To favor the passage through the sloped section of the interaction seam, we follow ref 83 in setting the velocities of the initial conditions to zero. Without an initial coherent superposition, this appears as a proper candidate to compare TSH-PFMi and TSH-EDC, see Section 2.7. We replicate the computational approach of the AIMS reference calculation,^83^ as detailed in Section 4.2, and calculate the first 50 fs of the TSH dynamics after the *S*_1_ excitation using 1000 trajectories for the TSH-PFMi, with η = 10^–4^, TSH-EDC, and TSH-ND methods, out of which, 976, 970, and 975, respectively conserve energy to a deviation of less than 1.0 eV so that they are included in the ensemble average.

In Figure 6b, we show the adiabatic *S*_1_ populations and fractions of trajectories obtained from the TSH calculations in comparison to the AIMS result digitized from Ibele et al.^83^ For the TSH simulations, we additionally show in Figure 6a the real part of the off-diagonal electronic density matrix element, depicting the coherent signature of the passages through the conical intersection seam. Looking first at the adiabatic populations, the AIMS reference^83^ predicts almost complete depopulation of *S*_1_ to *S*_0_ between 12 and 15.5 fs, followed by a back transfer of population to *S*_1_ of about 0.15 between 16 and 25 fs, before the third passage through the intersection again depopulates *S*_1_ almost entirely between 29 and 34 fs, after which the fourth passage through the interaction region slightly repopulates *S*_1_ from 35 fs onward. All TSH methods predict a qualitatively similar behavior. However, *S*_1_ always retains a residual population of at least 0.10. Further, the *S*_0_–*S*_1_ back-transfer of population appears to be much stronger. Between 16 and 25 fs one observes *S*_1_ population of up to 0.42 for TSH-PFMi, 0.48 for TSH-EDC, and up to 0.55 for TSH-ND. After 35 fs, one has up to 0.19 *S*_1_ population with TSH-PFMi, 0.24 with TSH-EDC, and up to 0.43 with TSH-ND. The TSH-PFMi and TSH-EDC simulations are internally consistent, while TSH-ND looses this property after the first back transfer. Although the TSH-PFMi and TSH-EDC predictions are quite similar, the TSH-PFMi result agrees slightly better with the AIMS reference.

**Figure 6:**
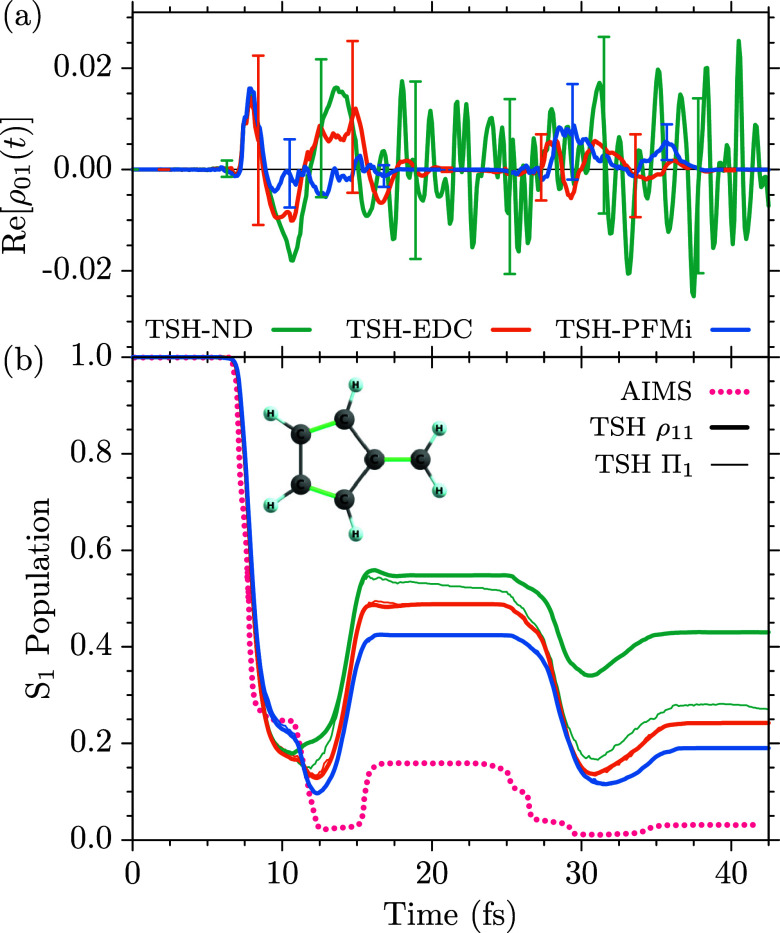
Nonadiabatic dynamics in fulvene after complete excitation into the S_1_ state with zero velocity. (a) Evolution of the *S*_0_–*S*_1_ electronic coherence, represented by the real part of the off-diagonal electronic density matrix element, Re­[ρ_01_(*t*)], obtained with TSH-ND (turquoise), TSH-EDC (orange), and TSH-PFMi (blue) using η = 10^–4^, respectively averaged over 975, 970, and 976 trajectories. Error bars indicate the 95% confidence intervals of the TSH averages. (b) Averaged adiabatic *S*_1_ population ρ_11_ (thick) and fraction of active trajectories Π_1_ (thin), see eq 50, same colors as in (a), compared with the results of an AIMS simulation (red, dotted) digitized from Ibele et al.^83^

Note that similar discrepancies between different TSH methods and AIMS results have been reported in the literature,^47,83,112^ which reassures that the TSH-PFMi calculations predict the same physics as established TSH methods in this difficult system. In fact it has been shown that one can obtain considerably better agreement with the AIMS reference, if one employs kinetic-energy rescaling along the NAC vector after surface hops in TSH-EDC.^83^

The coherences obtained from the TSH simulations are depicted in Figure 6a. The respective AIMS data is not available in the literature. Although the coherences are in general very small and the confidence intervals appear to be of the same order of magnitude, one can see that TSH-EDC and TSH-PFMi predict the emergence of coherences only between 7 and 20 fs, i.e., the first two passages through the intersection seam, and between 25 and 38 fs, i.e., the last two passages through the intersection seam, whereas there is no coherence when the wave packet is not close to the intersection seam. In contrast, TSH-ND, without decoherence, predicts coherent oscillations that last forever after the first passage through the intersection seam, similar to the case of IBr, Figure 3a. In any case, the results for the dynamics through the sloped *S*_1_/*S*_0_ intersection in fulvene show that TSH-PFMi performs well in such a difficult case, even providing a slightly better agreement with the AIMS reference than TSH-EDC.

### 5.3 Glycine

In Figures 7 and 8, we compare the results for the nonadiabatic dynamics following the initial coherent superposition of the lowest two adiabatic excited states of the neutral glycine molecule, $\Psi (0)=\frac{1}{\sqrt{2}}[\Psi_{1}(0)+\Psi_{2}(0)]$ that we obtained with the TSH-PFMi, with η = 10^–4^, TSH-EDC, and TSH-ND methods. For every TSH approach, we propagated the first 100 fs of the dynamics after the initial coherent superposition using 1000 trajectories, see Section 4.2 for details. After exclusion of those trajectories violating energy conservation by more than 1.0 eV and stochastically balancing the ensemble, as described in Section 3, we respectively retain 816 (TSH-PFMi), 824 (TSH-EDC), and 745 (TSH-ND) trajectories in the ensemble averages.

**Figure 7:**
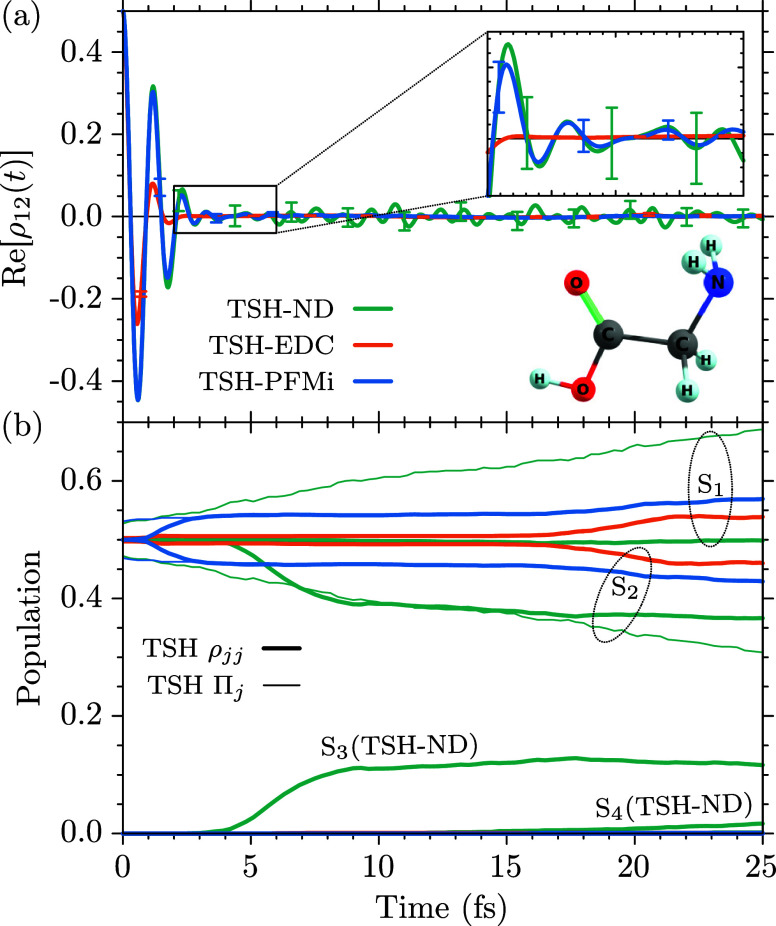
First 25 fs of the nonadiabatic dynamics in the I_*p*_ conformer of glycine induced by the symmetric superposition of the *S*_1_ and *S*_2_ excited states, $\Psi (0)=\frac{1}{\sqrt{2}}[\Psi_{1}(0)+\Psi_{2}(0)]$ . Results obtained with TSH-ND (turquoise), TSH-EDC (orange), and TSH-PFMi (blue), using η = 10^–4^, averaged over 745, 824, and 816 trajectories, respectively. (a) *S*_1_-*S*_2_ coherence, represented by the real part of the off-diagonal density matrix element, Re­[ρ_12_(*t*)]. The inset enhances the first 2−6 fs of the dynamics. (b) Averaged adiabatic populations ρ_*jj*_ (thick) and fractions of active trajectories Π_*j*_ (thin), see eq 50, using the same style and colors as in (a). The groups of curves corresponding to the S_1_ and S_2_ states are encircled.

**Figure 8:**
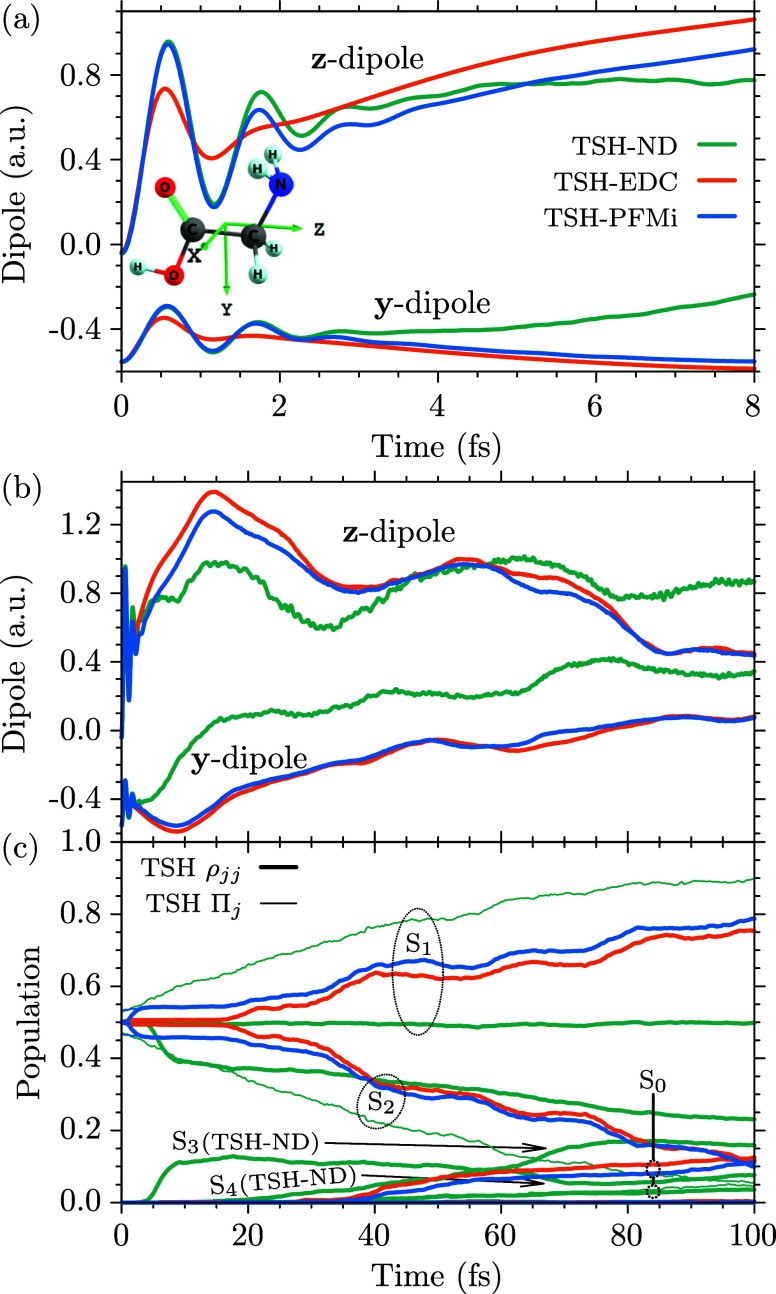
Nonadiabatic dynamics in the I_*p*_ conformer of glycine induced by the symmetric superposition of the *S*_1_ and *S*_2_ excited states, $\Psi (0)=\frac{1}{\sqrt{2}}[\Psi_{1}(0)+\Psi_{2}(0)]$ , resulting from the same TSH simulations as in Figure 7. (a) First 8 fs of the evolution of the *z* and *y* (in-plane) dipole components. The out-of-plane *x* dipole component remains close to zero and is not shown. (b) Same as (a) but for the full propagation time of 100 fs. (c) Adiabatic populations ρ_*jj*_ (thick) and fractions of active trajectories Π_*j*_ (thin). Same as in Figure 7b but extended up to 100 fs. The arrows connect the short-term and long-term parts of the respective *S*_3_ and *S*_4_ adiabatic populations that are only significant in the TSH-ND results.

In Figure 7, we compare the short-time behavior of the coherences, represented by the off-diagonal density matrix element, Re­[ρ_12_(*t*)], and the adiabatic populations obtained with the TSH-PFMi method, see Figure 2, with the corresponding TSH-ND and TSH-EDC results. The TSH-PFMi and TSH-ND methods predict coherent oscillations until about 5.0 fs of the dynamics, although the confidence intervals are considerably larger in the latter case. This is a consequence of the lacking decoherence in TSH-ND, due to which the coherences on the individual trajectories live forever. TSH-EDC predicts a much more rapid decay of the initial coherence, reaching zero at about 2.5 fs.

The population dynamics obtained with the TSH-PFMi and TSH-EDC approaches are internally consistent and show a similar behavior in that we observe a small *S*_2_ → *S*_1_ population transfer of a few percent starting after 17 fs, indicating passage through a weak NAC region. The populations obtained from the TSH-ND simulations, however, behave quite different. While the *S*_1_ state keeps a rather constant electronic population during this time frame, the *S*_2_ state transfers population into the *S*_3_ state between 4 and 10 fs, which is further transferred to *S*_4_ from 20 fs onward. The fractions of active trajectories, however, show a distinct character, in part owing to the fact that surface hops to the *S*_3_ and *S*_4_ surfaces are mostly forbidden by energy conservation (frustrated). The fraction of active trajectories in the *S*_2_ state shows a linear decrease, while it increases for *S*_1_. Internal consistency is not achieved with the TSH-ND method and the fraction of active trajectories behaves quite different from the TSH-EDC and TSH-PFMi results.

Finally, we discuss the implications of the initial coherent superposition of electronic states on the charge dynamics. A measure of charge dynamics can be provided by the evolution of the molecular electric dipole. They are shown in Figure 8, at short (panel a) and long (panel b) times, for the three respective TSH methods. In the *z*-component, the TSH-ND and TSH-PFMi approaches predict very similar coherent oscillations until about 4 fs, whereas the TSH-EDC result shows a stronger damped oscillation that lasts until about 2.1 fs. After 4 fs, the TSH-EDC and TSH-PFMi results show a similar rising character, whereas the TSH-ND dipole stays more or less constant. In the *y*-component, the behavior is similar, but the oscillations are of smaller amplitude. The *x*-component of the dipole is not shown since it is always very close to zero in this choice of coordinates and dynamics under study. In the case of the *z*-component, the dipole passes from almost zero to almost 1 a.u. in approximately 1 fs, i.e., an increase of almost 2.5 D, which is more than twice the dipole of HCl.^113^ This huge increase cannot be due to a significant change in the atomic positions in such a short time but must be the consequence of a strong reorganization of the electron density. Note that these oscillations are sensitive to the initial phases in the coherent superposition as demonstrated in Appendix [Sec secA.2].

On the longer time scale until 100 fs, the dipole moments and adiabatic populations obtained with the TSH-PFMi and TSH-EDC approaches agree very well, however, the TSH-ND results differ considerably. In particular, the TSH-ND population dynamics does not capture the stepwise *S*_2_ → *S*_1_ population transfer obtained with the TSH-PFMi and TSH-EDC approaches. Instead, TSH-ND predicts the *S*_1_ population to remain constant, while the *S*_2_ population is transferred to the *S*_3_ and *S*_4_ states. Further, the TSH-ND populations, as opposed to the TSH-EDC and TSH-PFMi ones, are not internally consistent. Since the dipole moment is evaluated taking into account the electronic coefficients, see Section 2.4, the rather strong differences in the population dynamics obtained with (TSH-PFMi, TSH-EDC) and without decoherence correction (TSH-ND) translate directly into the dipole expectation values, explaining the differences.

## 6 Conclusions

In this work, we have outlined a trajectory surface hopping approach tailored for nonadiabatic molecular dynamics after an initial coherent superposition of electronic states, as can be created by broad-band few-femtosecond UV and attosecond XUV pulses. To this end, we have introduced the projected forces and momenta (PFM) decoherence correction. It estimates the decoherence rate based on differences of the auxiliary active-potential and inactive-potential trajectory-velocity-projected forces and momenta, as well as an overall width of the wave packet, which we expressed by the geometrically averaged normal-mode frequencies. To use the PFM decoherence in fewest-switches TSH simulations, we devised an approach for the propagation of the auxiliary momenta based on the trajectory-velocity-projected forces, coining the total protocol TSH-PFM.

First, we have shown for the IBr and glycine molecules that the overall results are rather stable with respect to the detailed settings of the auxiliary momentum propagation. We then scrutinized the TSH-PFM method by comparing with results from high-level fully dimensional nonadiabatic dynamics calculations with the direct dynamics variational multiconfigurational Gaussian and ab initio multiple spawning methods, for an initial coherent superposition in the BMA­[5,5] and para-xylene molecules and for the dynamics through a sloped conical intersection in fulvene. Our results show that the TSH-PFM approach yields, for these systems, a balanced description of the respective dynamics that agrees well with the high-level references within the inherent limitations of the TSH method.

We also performed calculations for an initial coherence in the glycine molecule without a quantum reference, and, to put the TSH-PFM simulations into perspective, conducted conventional TSH simulations without decoherence correction (TSH-ND) and with the energy-based decoherence correction (TSH-EDC) for all systems. For the cases with an initial coherent superposition, we find that the TSH-PFM results are at short times reminiscent of the TSH-ND results, albeit exhibiting a slightly faster decoherence, while they agree with the TSH-EDC results at longer times after the decay of the initial coherence. This might reflect that the EDC has been designed for initial conditions containing a narrow distribution of total energies, at variance with those created by broad-band laser excitation, representing a new paradigm for the application of TSH and other nonadiabatic dynamics methods.

For glycine, we show that the signature of the initial coherent superposition of the two lowest excited states survives for as long as 4 fs as coherent oscillations in the molecular dipole, which changes rather dramatically from 0 to more than 2 D within 1 fs. This effect is captured as well with the TSH-ND method and, to a lesser extend, with TSH-EDC. At long times, however, the TSH-PFM and TSH-EDC results agree in the prediction of the dipole moment induced by nuclear motion, whereas the TSH-ND approach does not predict the proper populations, which is reflected in the dipole.

The use of the TSH-PFM method does not imply a significant increase of the computational effort with respect to the existing TSH approaches, so it is appropriate to study coupled electron and nuclear dynamics in molecules as large as those already accessible by these approaches. As irradiation of molecules with recently available attosecond XUV/X-ray pulses and few-fs UV pulses, due to their broad bandwidth, always generates a coherent superposition of electronic states, either in molecular cations or neutral molecules, the present approach can open new ways to theoretically investigate the early stages of molecular dynamics, which have remained so far unexplored.

## Supplementary Material



## Figures and Tables

**A1 fig9:**
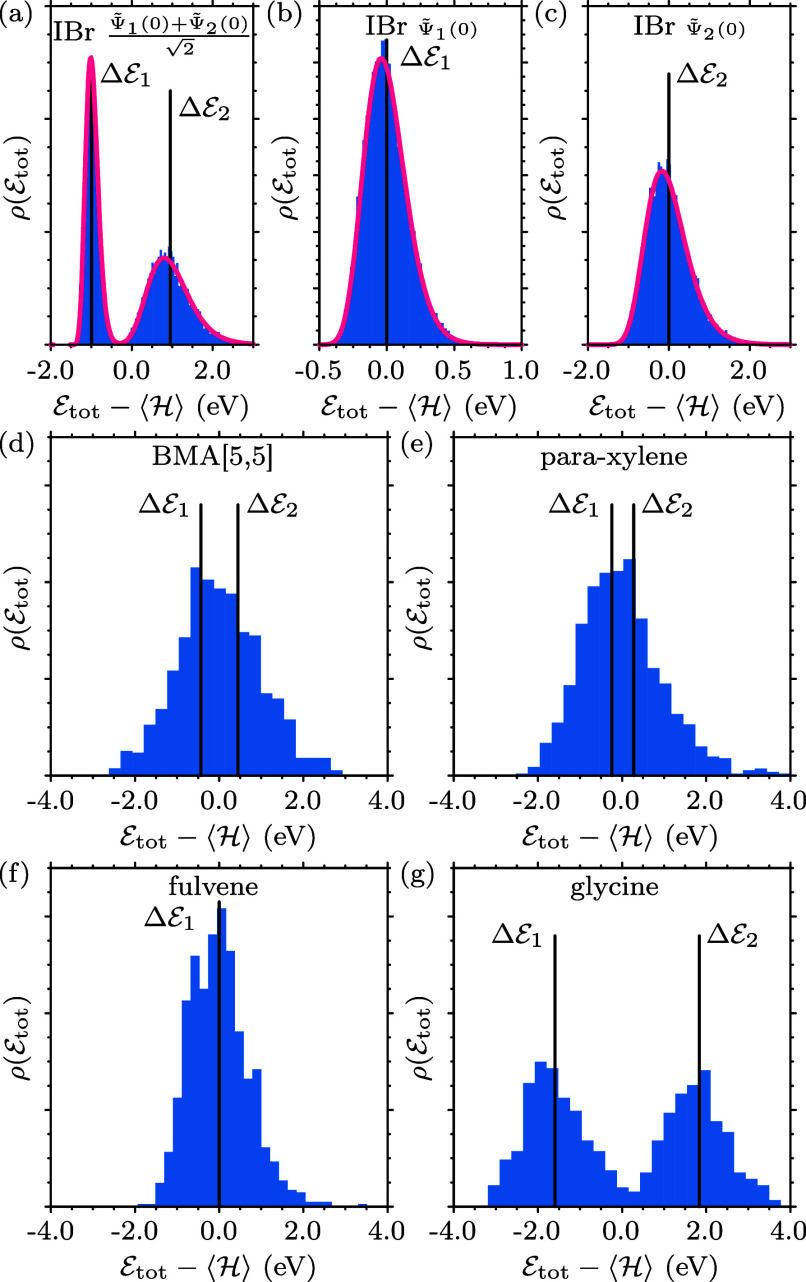
Histograms for the initial, *t* = 0, distributions of total energies centered around the expectation value, $E_{\mathrm{tot}}-\langle H\rangle$ , corresponding to the TSH-FMi (IBr), and TSH-PFMi (BMA­[5,5], para-xylene, fulvene, and glycine) results discussed in Section 5. The black lines indicate the relative positions of the averaged potential energies of the initially occupied states obtained with respect to the total energy expectation value as $\Delta E_{j}=\langle E_{j}\rangle +\langle T\rangle -\langle H\rangle$ . For the IBr molecule, the respective quantum distributions of total energies are given in red.

**A2 fig10:**
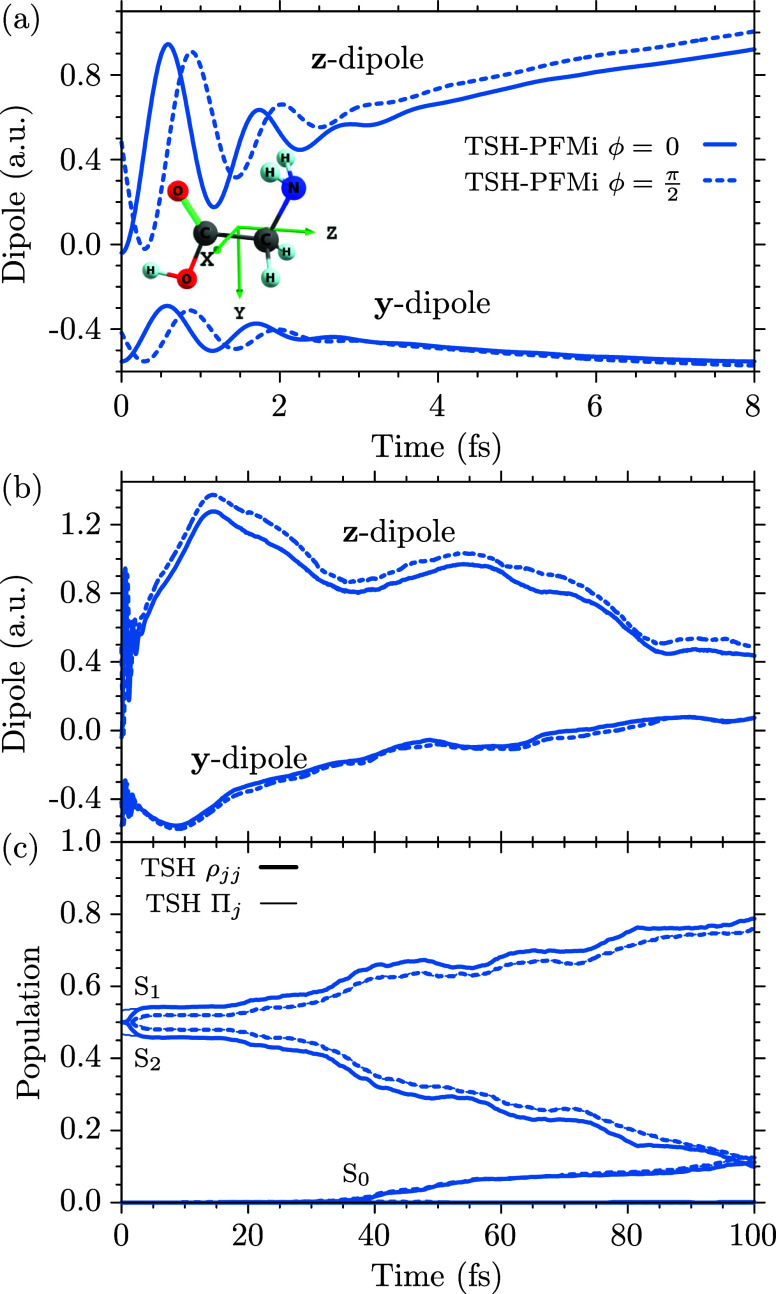
TSH-PFMi results for the nonadiabatic dynamics in the I_*p*_ conformer of glycine induced by the *S*_1_–*S*_2_ coherent superposition without a relative phase, φ = 0, $\Psi (0)=\frac{1}{\sqrt{2}}[\Psi_{1}(0)+\Psi_{2}(0)]$ , solid blue lines, shown already in Figure 8, compared to TSH-PFMi results obtained with a relative phase of $\varphi =\frac{\pi }{2}$ , i.e., $\Psi (0)=\frac{1}{\sqrt{2}}[\Psi_{1}(0)+i\Psi_{2}(0)]$ , dashed blue lines. (a) First 8 fs of the evolution of the *z* and *y* (in-plane) dipole components. (b) Continuation of (a) until 100 fs. The out-of-plane *x* dipole component remains close to zero and is not shown. (c) Adiabatic populations ρ*_jj_* (thick) and fractions of active trajectories Π_*j*_ (thin). The TSH-PFMi results have been averaged over 816, φ = 0, and 823 trajectories, $\varphi =\frac{\pi }{2}$ , respectively.

## APPENDIX

### A.1 Total Energy Distributions

Figure 9 depicts the distributions of total energies at the start, *t* = 0, of the TSH-FMi (IBr) and TSH-PFMi (BMA­[5,5], para-xylene, fulvene, and glycine) simulations discussed in Section 5. The histograms comprise the respective valid TSH-FMi/PFMi trajectories, i.e., 10^4^ for IBr, 1000 for BMA­[5,5] and para-xylene, 976 for fulvene and 816 for glycine. For an initial condition, {***R***_ν_(0), ***P***_ν_(0)}, the total energy is obtained as

$$E_{\mathrm{tot},\nu }(0)=\overset{T_{\nu }(0)}{\overset{︷}{\frac{1}{2}P_{\nu }^{T}(0)M^{-1}P_{\nu }(0)}}+E_{a_{\nu }(0)}(R_{\nu }(0))$$
A1

The first term represents the kinetic energy, *T*_ν_(0), determined by the initial momentum, ***P***_ν_(0), and mass matrix, ***M***; the second term is the potential energy of the stochastically-determined initial active potential, *a*_ν_(0), at the configuration ***R***_ν_(0). The expectation value is obtained as the respective average, $\left\langle H(0)\rangle =\frac{1}{N_{\mathrm{IC}}}\underset{\nu }{\sum }E_{\mathrm{tot},\nu }(0\right)$ . Note that the total-energy distributions obtained for the TSH-ND, TSH-EDC, and TSH-FM/PFM simulations are expected to be very similar, since they only differ in the, respectively, independent stochastic selection of the initial active potentials.

For the IBr molecule, the energy distributions of the respective initial states of the quantum simulation are given in red. They have been obtained as the real part of the Fourier transformation of the time autocorrelation function of the numerical wave function. Dropping the ‘tot’ subscript for brevity, one has

|Ψ(E,0)|2∝Re[F−1{⟨Ψ(0)|Ψ(t)⟩}(E)]
A2

while the respective energy expectation values have been obtained as $\langle H(0)\rangle =\int |\Psi (E,0){|}^{2}EdE$ , for normalized wave functions, 1 = ∫ |Ψ­( E ,0)|^2^ *d* E .

The relative positions of the average potential energies of the initially-occupied states, $\Delta E_{j}$ , are indicated in Figure 9 as well. The comparison of the total-energy distributions obtained for the initial coherent superpositions in IBr, Figure 9a, BMA­[5,5], Figure 9d, para-xylene, Figure 9e, and glycine, Figure 9g, shows that only for IBr and glycine one obtains a bipartite distribution of total energies. In these cases, the initial potential-energy difference is about 1.9 eV for IBr and 3.4 eV for glycine, larger than the respective widths of the total energy distributions on the respective electronic potentials. The potential-energy differences for BMA­[5,5], 0.9 eV, and para-xylene, 0.5 eV, are small enough that the total-energy distribution maintains a single-peak characteristic.

### A.2 Phase Dependence

Here we exemplify, for the I_*p*_-conformer of glycine, the influence of different relative phases between the states in the coherent superposition onto the non-adiabatic dynamics predicted with the TSH-PFMi approach. To this end we repeated the TSH-PFMi calculation detailed in Section 5.3 for the same set of 1000 initial positions and momenta, using a coherence with a relative phase of $\varphi =\frac{\pi }{2}$ , $\Psi (0)=\frac{1}{\sqrt{2}}[\Psi_{1}(0)+i\;\Psi_{2}(0)]$ . Out of the 1000 trajectories, which were started using an independent stochastic selection of the initial active potentials based on the adiabatic *S*_1_ and *S*_2_ populations, we obtained 823 balanced valid trajectories with a total energy conservation threshold of Δ E _tot_ ≤ 1.0 eV, see Section 3.

In Figure 10 we compare the so-obtained trajectory averages for the dipole expectation values and population dynamics with the respective results without an initial phase, φ = 0, that have been shown in the discussion of glycine in Figures 7 and 8. The zoom into the fast oscillations of the dipole moments in Figure 10a shows that the phase from the initial coherence is directly reflected in the observable as a phase shift of $\frac{\pi }{2}$ between the curves corresponding to the φ = 0 and $\varphi =\frac{\pi }{2}$ initial coherent wave packets. This early part of the dipole moment evolution is thus sensitive to the relative coherent phases. On the contrary, the initial phases neither considerably affect the long-term behavior of the dipole moments, Figure 10b, nor the population dynamics, Figure 10c. The observed small differences are most likely due to the, respectively, independent stochastic selection of the initial active potentials employed for both sets of trajectories.

## Notes

Protocol to construct the diabatization matrix; data on the energy conservation for all molecules; and optimized ground-state molecular geometries and corresponding harmonic normal-mode frequencies for all molecules but IBr, obtained as described in Section 4.2(PDF)
