# Supplementary material for: Modeling the Evolution of Laser-Induced Electronic Coherences with Trajectory Surface Hopping
Source: J Chem Theory Comput. 2025 Oct 20;21(21):10645–68. doi: 10.1021/acs.jctc.5c00531 (PMC12613326; doi:10.1021/acs.jctc.5c00531)
Supplement: Supplementary file 1 [file ct5c00531_si_001.pdf]

Supplementary Material for: Modeling the evolution of laser-induced electronic coherences with trajectory surface hopping

Gilbert Grell,[*,†,‡] Jesús González-Vázquez,[‡] Francisco Fernández Villoria,[†,‡] Alicia Palacios,[‡,¶] and Fernando Martín[*,†,‡]

†*Instituto Madrileño de Estudios Avanzados en Nanociencia (IMDEA), Madrid, 28049, Spain*

‡*Departamento de Química, Módulo 13, Universidad Autónoma de Madrid, Madrid, 28049, Spain*

¶*Institute for Advanced Research in Chemical Sciences (IAdChem), Universidad Autónoma de Madrid, Madrid, 28049, Spain*

E-mail: gilbert.grell@imdea.org; fernando.martin@uam.es

September 18, 2025

S1 Local diabatization

Let $\boldsymbol{S}(t_0 + \Delta t)$ be the overlap matrix between the adiabatic states at the beginning and end of the nuclear time step $[t_0, t_0 + \Delta t]$,

$$S_{ij}(t_0 + \Delta t, t_0) = \langle \Psi_i(t_0 + \Delta t) | \Psi_j(t_0) \rangle \tag{S1}$$

Assuming that N_p electronic potentials are propagated, its column vectors, $\boldsymbol{s}_k = (S_{1k}(t_0 + \Delta t, t_0), ..., S_{N_p k}(t_0 + \Delta t, t_0))^T$, contain the expansion of the adiabatic states at the beginning of the time step in terms of the states at the end,

$$\Psi_k(t_0) = \sum_j S_{jk}(t_0 + \Delta t, t_0) \Psi_j(t_0 + \Delta t) \,, \tag{S2}$$

$$= \boldsymbol{s}_k^T \cdot \boldsymbol{\Psi}(t_0 + \Delta t) \,. \tag{S3}$$

In the last step we have arranged the adiabatic states at the end of the time step as a vector, $\boldsymbol{\Psi}(t_0 + \Delta t) = (\Psi_1(t_0 + \Delta t), ..., \Psi_{N_p}(t_0 + \Delta t))^T$. If the overlap matrix, Eq. (S1), is unitary, Eq. (S2) defines the local diabatization,[1,2] since the local diabatic wave functions coincide with the adiabatic ones at the beginning of the time step, $\Psi_j(t_0) = \tilde{\Psi}_j(t_0)$, and the diabatic wave functions should be constant over the time step, i.e., $\tilde{\Psi}_j(t_0 + \Delta t) = \tilde{\Psi}_j(t_0)$. In most simulations, however, this cannot be achieved since the Hilbert space spanned by the adiabatic wave functions can change between the different calculations at t_0 and $t_0 + \Delta t$, as one can only afford to include a limited number of states into the simulation. As a consequence, the overlap matrix in Eq. (S1) looses its unitarity and one has to construct a norm-conserving unitary transformation. A common way to achieve this is the Löwdin orthogonalization.[1] However, it can become unstable in case an electronic potential of different character enters the manifold of propagated electronic potentials at $t_0 + \Delta t$, leading to a row of very small values in the overlap matrix.

We use a locally modified version of the SHARC trajectory surface hopping (TSH) code,[3]

into which we have implemented the TSH-FM and TSH-PFM algorithms described in the main text. The SHARC code[3] circumvents the aforementioned problem by constructing the local diabatization matrix $\boldsymbol{U}(t_0, t_0 + \Delta t)$ for a single nuclear time step, $[t_0, t_0 + \Delta t]$, such that the overlap $\langle\tilde{\Psi}_j(t_0 + \Delta t)|\Psi_j(t_0)\rangle$ is maximized for every state j.

First, a projection matrix for the first electronic state, $\Psi_1(t_0)$, is constructed as the dyadic product of the corresponding first column of the overlap matrix,

$$\boldsymbol{P}^1 = \boldsymbol{s}_1 \boldsymbol{s}_1^T \,, \tag{S4}$$

$$P_{ij}^1 = S_{i1}(t_0 + \Delta t, t_0) S_{j1}(t_0 + \Delta t, t_0) \,, \tag{S5}$$

where we have used the realness of the overlap matrix, $S_{ij}(t_0 + \Delta t, t_0)^* = S_{ij}(t_0 + \Delta t, t_0)$. The projection matrix $\boldsymbol{P}^1$ is then diagonalized. Since $\mathrm{rank}(\boldsymbol{P}^1) = 1$, it has only one nonzero eigenvalue, i.e., the squared norm of its constructing vector, $\lambda_1 = ||\boldsymbol{s}_1||^2$, while the corresponding eigenvector is the respectively normalized $\boldsymbol{s}_1$,

$$\boldsymbol{P}^1 = \underbrace{\left(\boldsymbol{u}_1^1, ..., \boldsymbol{u}_{N_p}^1\right)}_{\boldsymbol{U}^1} \mathrm{diag}\{||\boldsymbol{s}_1||^2, 0, ..., 0\} \underbrace{\left(\boldsymbol{u}_1^{1^T}, ..., \boldsymbol{u}_{N_p}^{1\ T}\right)^T}_{\boldsymbol{U}^{1^T}} \,, \tag{S6}$$

$$\boldsymbol{u}_1^1 = \frac{\boldsymbol{s}_1}{||\boldsymbol{s}_1||} \,, \tag{S7}$$

$$\boldsymbol{P}^1 \cdot \boldsymbol{u}_1^1 = (\boldsymbol{s}_1 \boldsymbol{s}_1^T) \cdot \frac{\boldsymbol{s}_1}{||\boldsymbol{s}_1||} = \frac{\boldsymbol{s}_1}{||\boldsymbol{s}_1||} \boldsymbol{s}_1^T \boldsymbol{s}_1 = \boldsymbol{u}_1^1 ||\boldsymbol{s}_1||^2 \,. \tag{S8}$$

Since the projector is symmetric, $\boldsymbol{U}^1$ is unitary and $\boldsymbol{u}_1^1$ and the other unit vectors form an orthonormal basis. SHARC[3] takes the vector $\boldsymbol{u}_1^1$ as the diabatization vector for the first electronic potential, which maximizes the overlap,

$$\tilde{\Psi}_1(t_0 + \Delta t) = \boldsymbol{u}_1^{1^T} \boldsymbol{\Psi}(t_0 + \Delta t) \,, \tag{S9}$$

$$\langle\tilde{\Psi}_1(t_0 + \Delta t)|\Psi_1(t_0)\rangle = \boldsymbol{u}_1^{1^T} \boldsymbol{s}_1 = ||\boldsymbol{s}_1|| \tag{S10}$$

The other diabatization vectors are constructed similarly as follows:

1. For the second state, rotate the overlap matrix with the previous unitary transformation matrix,

$$\boldsymbol{S}^2(t_0+\Delta t, t_0) = \boldsymbol{U}^{1^T}\boldsymbol{S}(t_0+\Delta t, t_0) \tag{S11}$$

$$= \begin{pmatrix} \boldsymbol{u}_1^{1^T}\boldsymbol{s}_1 & \boldsymbol{u}_1^{1^T}\boldsymbol{s}_2 & \cdots \\ \boldsymbol{u}_2^{1^T}\boldsymbol{s}_1 & \boldsymbol{u}_2^{1^T}\boldsymbol{s}_2 & \cdots \\ \vdots & & \\ \boldsymbol{u}_{N_p}^{1}{}^T\boldsymbol{s}_1 & \boldsymbol{u}_{N_p}^{1}{}^T\boldsymbol{s}_2 & \cdots \end{pmatrix} = \begin{pmatrix} ||\boldsymbol{s}_1|| & \overbrace{\boldsymbol{u}_1^{1^T}\boldsymbol{s}_2}^{\approx 0} & \overbrace{\cdots}^{\approx 0} \\ 0 & \boldsymbol{u}_2^{1^T}\boldsymbol{s}_2 & \cdots \\ \vdots & & \\ 0 & \boldsymbol{u}_{N_p}^{1}{}^T\boldsymbol{s}_2 & \cdots \end{pmatrix}. \tag{S12}$$

Where we have used Eq. (S10), and the orthonormality of the vectors $\boldsymbol{u}_j^1$. Since $\boldsymbol{u}_1^1 = \frac{\boldsymbol{s}_1}{||\boldsymbol{s}_1||}$, one has,

$$\boldsymbol{u}_i^{1^T}\boldsymbol{u}_j^1 = \delta_{ij}\,, \tag{S13}$$

$$(\boldsymbol{u}_{j>1}^1)^T\boldsymbol{s}_1 = 0\,. \tag{S14}$$

Further, the elements $\boldsymbol{u}_1^{1^T}\boldsymbol{s}_{j>1} \approx 0$ are expected to be small, as the overlap matrix should not deviate far from unitarity. Note that $\boldsymbol{S}^2(t_0+\Delta t, t_0)$ is the overlap between the $\boldsymbol{U}^1$-rotated electronic states at the end of the time step, $\boldsymbol{\Psi}^1(t_0+\Delta t) = \boldsymbol{U}^{1^T}\boldsymbol{\Psi}(t_0+\Delta t)$, along columns and the original states at the beginning, $\boldsymbol{\Psi}(t_0)$, along rows.

2. Remove the first row and column of $\boldsymbol{S}^2(t_0+\Delta t, t_0)$ and continue with the $N_p - 1$ dimensional sub matrix,

$$\tilde{\boldsymbol{S}}^2(t_0+\Delta t, t_0) = \begin{pmatrix} \boldsymbol{u}_2^{1^T}\boldsymbol{s}_2 & \boldsymbol{u}_2^{1^T}\boldsymbol{s}_3 & \cdots \\ \vdots & \vdots & \\ \boldsymbol{u}_{N_p}^{1}{}^T\boldsymbol{s}_2 & \boldsymbol{u}_{N_p}^{1}{}^T\boldsymbol{s}_3 & \cdots \end{pmatrix}. \tag{S15}$$

3. Construct the $N_p - 1$ dimensional projection matrix for the second state in terms of the rotated basis $\boldsymbol{\Psi}^1(t_0+\Delta t)$ without the first diabatic state, $\tilde{\Psi}_1(t_0+\Delta t)$. Taking the

first column vector of $\tilde{\boldsymbol{S}}^2(t_0+\Delta t, t_0) = \left(\tilde{\boldsymbol{s}}_2, ..., \tilde{\boldsymbol{s}}_{N_p}\right)$, one has

$$\boldsymbol{P}^2 = \tilde{\boldsymbol{s}}_2 \tilde{\boldsymbol{s}}_2^T \in \mathbb{R}^{N_p-1 \times N_p-1} \,, \tag{S16}$$

with $\tilde{\boldsymbol{s}}_j = \left(\boldsymbol{u}_2^{1^T} \boldsymbol{s}_j, ..., \boldsymbol{u}_{N_p}^{1\ \ T} \boldsymbol{s}_j\right)^T$.

4. Following Eqs. (S6)-(S10), $\boldsymbol{P}^2$ is diagonalized, which yields the N_p-1 dimensional unitary transformation matrix, $\tilde{\boldsymbol{U}}^2 = \left(\tilde{\boldsymbol{u}}_2^2, ..., \tilde{\boldsymbol{u}}_{N_p}^2\right)$, and the eigenvalues $\text{diag}\{||\tilde{\boldsymbol{s}}_2||^2, 0, ..., 0\}$. Enlarging the dimension of the eigenvector, $\tilde{\boldsymbol{u}}_2^2$, corresponding to the nonzero eigenvalue, $||\tilde{\boldsymbol{s}}_2||^2$, by adding a zero in the first dimension, allows to express $\tilde{\Psi}_2(t_0+\Delta t)$ in terms of the original adiabatic basis and obtain the overlap with $\Psi_2(t_0)$,

$$\boldsymbol{u}_2^2 = \left(0, U_{22}^2, ..., U_{N_p 2}^2\right)^T = \left(0, (\tilde{\boldsymbol{u}}_2^2)^T\right)^T \,, \tag{S17}$$

$$\tilde{\Psi}_2(t_0+\Delta t) = \boldsymbol{u}_2^{2^T} \boldsymbol{\Psi}^1(t_0+\Delta t) \,, \tag{S18}$$

$$= \boldsymbol{u}_2^{2^T} \boldsymbol{U}^{1^T} \boldsymbol{\Psi}(t_0+\Delta t) \,, \tag{S19}$$

$$\langle \tilde{\Psi}_2(t_0+\Delta t) | \Psi_2(t_0) \rangle = \boldsymbol{u}_2^{2^T} \boldsymbol{U}^{1^T} \boldsymbol{s}_2 \,. \tag{S20}$$

5. Enlarge $\tilde{\boldsymbol{U}}^2$ to full (N_p) dimensionality by adding 1 on the diagonal, and zeros on the off-diagonal parts,

$$\boldsymbol{U}^2 = \begin{pmatrix} 1 & 0 & 0 & \cdots \\ 0 & U_{22}^2 & U_{23}^2 & \cdots \\ 0 & U_{32}^2 & U_{33}^2 & \cdots \\ \vdots & \vdots & \vdots & \ddots \end{pmatrix} . \tag{S21}$$

6. Evaluate the total rotation matrix until the second state,

$$\boldsymbol{U} = \boldsymbol{U}^1 \boldsymbol{U}^2 \,. \tag{S22}$$

7. Iterate points 1.-6. for the remaining $k = 3, .., N_p$ states, using the new rotation matrix $\boldsymbol{U} = \boldsymbol{U}^1\boldsymbol{U}^2 \cdots \boldsymbol{U}^{k-1}$ to rotate the overlap matrix,

$$\boldsymbol{S}^k(t_0 + \Delta t, t_0) = \boldsymbol{U}^{k-1^T} \cdots \boldsymbol{U}^{2^T}\boldsymbol{U}^{1^T}\boldsymbol{S}(t_0 + \Delta t, t_0)\,. \tag{S23}$$

Further, remove the first $k-1$ rows and columns from the overlap matrix before constructing the $N_p - (k-1)$ dimensional projector $\boldsymbol{P}^k$.

8. In the herein-employed SHARC code,[3] the local diabatization matrix is then obtained as the product of all iteratively obtained transformation matrices $\boldsymbol{U}^k$,

$$\boldsymbol{U}(t_0, t_0 + \Delta t) = \boldsymbol{U}^1 \cdots \boldsymbol{U}^{N_p}\,. \tag{S24}$$

$\boldsymbol{U}(t_0, t_0 + \Delta t)$ is unitary, since all matrices $\boldsymbol{U}^k$ are constructed to be unitary. The electronic states in the local diabatic basis at the end of the nuclear time step are obtained as,

$$\tilde{\boldsymbol{\Psi}}(t_0 + \Delta t) = \boldsymbol{U}(t_0, t_0 + \Delta t)^T\boldsymbol{\Psi}(t_0 + \Delta t)\,. \tag{S25}$$

S2 Conservation of total energy

Table 1: Number of balanced valid trajectories obtained as outlined in Sect. 3 of the main text, using $\Delta\mathcal{E}_{\text{tot}} \leq 0.1$, 0.2, 0.5, 1.0 eV. Results for the TSH-FMi/PFMi simulations of the dynamics induced by the initial coherent superpositions in all molecules but fulvene, where a total S_1 excitation with zero initial momenta has been studied. For details see main text.

	nr. of trajectories				
molecule	total	$\Delta\mathcal{E}_{\text{tot}} \leq 1.0$ eV	$\Delta\mathcal{E}_{\text{tot}} \leq 0.5$ eV	$\Delta\mathcal{E}_{\text{tot}} \leq 0.2$ eV	$\Delta\mathcal{E}_{\text{tot}} \leq 0.1$ eV
IBr	10^4	10^4	10^4	10^4	10^4
BMA[5,5]	1000	1000	1000	1000	1000
para-xylene	1000	1000	1000	1000	1000
fulvene	1000	976	968	954	941
glycine	1000	816	496	212	175

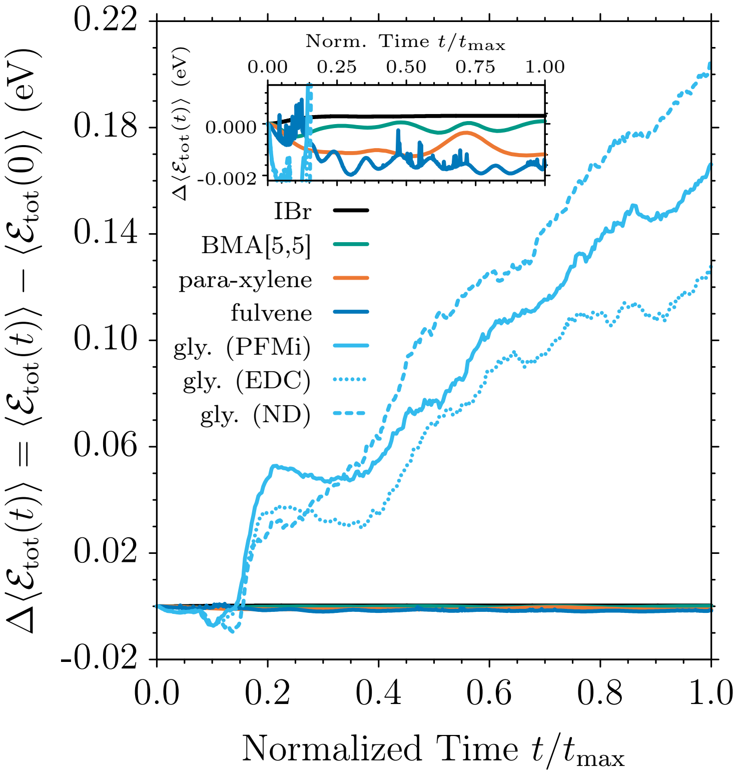

Figure S1: Deviation of the ensemble-averaged total energies from their $t = 0$ value for the TSH-FMi/PFMi simulations of the dynamics induced by the initial coherent superpositions in all molecules but fulvene, where a complete S_1 excitation with zero initial momenta has been studied. For glycine, the respective TSH-EDC and TSH-ND results are shown as well. The time axis has been normalized to the maximum durations shown in the main text, 110 fs, 15 fs, 15 fs, 42.5 fs, and 100 fs, respectively, for IBr, BMA[5,5], para-xylene, fulvene, and glycine. Taking, as in the main text, the $\Delta\mathcal{E}_{tot} \leq 1.0$ eV ensembles of balanced valid trajectories, 10^4, 10^3, 10^3, and 976 trajectories have been included in the ensemble average for IBr, BMA[5,5], para-xylene, and fulvene, respectively. For glycine, 745, 824, and 816 trajectories have, respectively, been employed for the TSH-ND, TSH-EDC, and TSH-PFMi results. For details see Sects. 4 and 5 of the main text.

S3 Molecular geometries and frequencies

S3.1 BMA[5,5]

Optimized geometry of BMA[5,5], obtained as described in the main text.

#	atom	x [a.u.]	y [a.u.]	z [a.u.]
1	C	5.57699758E-01	-5.16301247E-03	-3.97742245E+00
2	C	1.51667567E+00	-1.90639945E+00	-1.97106205E+00
3	C	3.54294215E+00	-1.93935591E-01	-9.00395055E-01
4	C	1.74682555E+00	1.76023880E+00	1.67209194E-01
5	C	5.52792181E-01	-4.85267229E-03	2.17184345E+00
6	C	-6.38040496E-01	-1.77014349E+00	1.65483090E-01
7	C	-2.43244970E+00	1.83925009E-01	-9.05183616E-01
8	C	-4.04476194E-01	1.89627889E+00	-1.97278803E+00
9	C	-4.94550253E+00	3.42842112E-01	-9.07194621E-01
10	C	6.05599498E+00	-3.52851140E-01	-8.98383383E-01
11	H	2.08066749E+00	6.79716517E-01	-5.16388750E+00
12	H	-9.63374186E-01	-6.90164299E-01	-5.16624435E+00
13	H	2.06032321E+00	-3.77151099E+00	-2.60162790E+00
14	H	2.52007809E+00	3.54207473E+00	7.98832408E-01
15	H	1.97546103E+00	-8.76004868E-01	3.36066528E+00
16	H	-8.71770770E-01	8.66417378E-01	3.35830827E+00
17	H	-1.41230261E+00	-3.55191553E+00	7.96048992E-01
18	H	-9.47114132E-01	3.76132662E+00	-2.60441118E+00
19	H	-5.90821127E+00	1.96655353E+00	-1.66411300E+00
20	H	-6.10628632E+00	-1.14658667E+00	-1.51975856E-01
21	H	7.21557276E+00	1.13665442E+00	-1.41465286E-01
22	H	7.01990969E+00	-1.97663842E+00	-1.65360234E+00

Harmonic normal mode frequencies of BMA[5,5], obtained as described in the main text.

mode	[cm^-1]	[eV]	[a.u.]
1	1.50071E+02	1.86064E-02	6.83772E-04
2	1.62982E+02	2.02072E-02	7.42601E-04
3	2.87674E+02	3.56671E-02	1.31074E-03
4	3.70298E+02	4.59111E-02	1.68720E-03
5	4.40980E+02	5.46746E-02	2.00925E-03
6	4.82711E+02	5.98485E-02	2.19939E-03
7	4.99156E+02	6.18875E-02	2.27432E-03
8	6.36203E+02	7.88792E-02	2.89876E-03
9	7.10682E+02	8.81133E-02	3.23810E-03
10	7.32730E+02	9.08470E-02	3.33856E-03
11	7.41361E+02	9.19170E-02	3.37789E-03
12	7.89939E+02	9.79400E-02	3.59923E-03
13	8.08310E+02	1.00218E-01	3.68293E-03
14	8.13806E+02	1.00899E-01	3.70798E-03
15	8.17576E+02	1.01367E-01	3.72515E-03
16	8.52855E+02	1.05741E-01	3.88589E-03
17	8.56251E+02	1.06162E-01	3.90137E-03
18	8.90143E+02	1.10364E-01	4.05579E-03
19	9.56650E+02	1.18609E-01	4.35882E-03
20	9.56762E+02	1.18623E-01	4.35933E-03
21	9.64975E+02	1.19642E-01	4.39675E-03
22	9.71277E+02	1.20423E-01	4.42546E-03
23	9.94188E+02	1.23264E-01	4.52986E-03
24	1.00851E+03	1.25040E-01	4.59513E-03
25	1.03388E+03	1.28185E-01	4.71072E-03
26	1.04703E+03	1.29816E-01	4.77064E-03
27	1.11728E+03	1.38525E-01	5.09068E-03
28	1.13573E+03	1.40812E-01	5.17476E-03
29	1.21067E+03	1.50105E-01	5.51624E-03
30	1.23342E+03	1.52925E-01	5.61989E-03
31	1.24391E+03	1.54225E-01	5.66766E-03
32	1.28567E+03	1.59403E-01	5.85796E-03
33	1.33102E+03	1.65025E-01	6.06457E-03
34	1.34323E+03	1.66540E-01	6.12023E-03
35	1.35859E+03	1.68443E-01	6.19018E-03
36	1.36235E+03	1.68910E-01	6.20732E-03
37	1.36645E+03	1.69418E-01	6.22601E-03
38	1.37868E+03	1.70935E-01	6.28175E-03
39	1.40060E+03	1.73652E-01	6.38161E-03
40	1.42601E+03	1.76802E-01	6.49736E-03
41	1.42871E+03	1.77137E-01	6.50967E-03
42	1.45478E+03	1.80370E-01	6.62849E-03

43	1.58295E+03	1.96261E-01	7.21244E-03
44	1.58716E+03	1.96782E-01	7.23162E-03
45	1.65688E+03	2.05428E-01	7.54932E-03
46	1.65880E+03	2.05665E-01	7.55806E-03
47	1.83011E+03	2.26905E-01	8.33861E-03
48	1.83632E+03	2.27675E-01	8.36689E-03
49	3.23333E+03	4.00882E-01	1.47322E-02
50	3.23362E+03	4.00918E-01	1.47335E-02
51	3.27835E+03	4.06463E-01	1.49372E-02
52	3.27969E+03	4.06631E-01	1.49434E-02
53	3.29387E+03	4.08388E-01	1.50080E-02
54	3.29538E+03	4.08575E-01	1.50148E-02
55	3.30436E+03	4.09689E-01	1.50558E-02
56	3.30861E+03	4.10216E-01	1.50752E-02
57	3.31624E+03	4.11161E-01	1.51099E-02
58	3.31705E+03	4.11261E-01	1.51136E-02
59	3.39246E+03	4.20611E-01	1.54572E-02
60	3.39259E+03	4.20627E-01	1.54578E-02

S3.2 Para-xylene

Optimized geometry of para-xylene, obtained as described in the main text.

#	atom	x [a.u.]	y [a.u.]	z [a.u.]
1	C	-2.77841992E-02	-5.57852084E-02	-2.70857812E+00
2	C	-2.29478393E+00	-1.91031851E-02	-1.34478712E+00
3	C	2.23941634E+00	-3.49306283E-02	-1.34478710E+00
4	C	-2.29467027E+00	1.34819347E-02	1.29119822E+00
5	C	2.23953002E+00	-2.34553005E-03	1.29119825E+00
6	C	-2.74696896E-02	3.43365880E-02	2.65498922E+00
7	C	-2.76297231E-02	-1.14874583E-02	-5.55580112E+00
8	C	-2.76243282E-02	-9.96067873E-03	5.50221226E+00
9	H	-4.08644352E+00	-3.24940396E-02	-2.35785526E+00
10	H	4.03093883E+00	-6.08291620E-02	-2.35785512E+00
11	H	-4.08619277E+00	3.93805119E-02	2.30426625E+00
12	H	4.03118961E+00	1.10453388E-02	2.30426639E+00
13	H	1.64161591E+00	-9.65811976E-01	-6.31442057E+00
14	H	-1.70349510E+00	-9.54138941E-01	-6.31442037E+00
15	H	-2.08570991E-02	1.92933858E+00	-6.27629367E+00
16	H	-1.69686871E+00	9.44366303E-01	6.26083136E+00
17	H	-3.43996951E-02	-1.95078647E+00	6.22270545E+00
18	H	1.64824227E+00	9.32688695E-01	6.26083144E+00

Harmonic normal mode frequencies of para-xylene, obtained as described in the main text.

mode	[cm^-1]	[eV]	[a.u.]
1	5.62793E+01	6.97775E-03	2.56428E-04
2	7.27756E+01	9.02303E-03	3.31590E-04
3	1.33473E+02	1.65486E-02	6.08149E-04
4	2.86360E+02	3.55042E-02	1.30475E-03
5	3.02279E+02	3.74779E-02	1.37729E-03
6	3.87780E+02	4.80786E-02	1.76685E-03
7	3.98330E+02	4.93866E-02	1.81492E-03
8	4.61595E+02	5.72305E-02	2.10318E-03
9	4.73805E+02	5.87443E-02	2.15881E-03
10	5.09370E+02	6.31539E-02	2.32086E-03
11	6.58790E+02	8.16796E-02	3.00167E-03
12	7.42229E+02	9.20248E-02	3.38185E-03
13	7.69898E+02	9.54552E-02	3.50791E-03
14	8.27853E+02	1.02641E-01	3.77198E-03
15	8.51029E+02	1.05514E-01	3.87757E-03
16	8.65037E+02	1.07251E-01	3.94140E-03
17	8.73791E+02	1.08336E-01	3.98128E-03
18	1.01678E+03	1.26065E-01	4.63281E-03
19	1.05399E+03	1.30678E-01	4.80231E-03
20	1.05887E+03	1.31283E-01	4.82456E-03
21	1.08285E+03	1.34256E-01	4.93383E-03
22	1.08967E+03	1.35102E-01	4.96489E-03
23	1.17136E+03	1.45230E-01	5.33709E-03
24	1.23681E+03	1.53345E-01	5.63531E-03
25	1.26644E+03	1.57019E-01	5.77035E-03
26	1.26960E+03	1.57411E-01	5.78474E-03
27	1.35884E+03	1.68475E-01	6.19132E-03
28	1.44406E+03	1.79041E-01	6.57964E-03
29	1.46538E+03	1.81684E-01	6.67675E-03
30	1.46925E+03	1.82163E-01	6.69438E-03
31	1.48692E+03	1.84355E-01	6.77491E-03
32	1.54374E+03	1.91399E-01	7.03378E-03
33	1.54812E+03	1.91943E-01	7.05377E-03
34	1.55155E+03	1.92368E-01	7.06940E-03
35	1.56251E+03	1.93727E-01	7.11934E-03
36	1.57989E+03	1.95882E-01	7.19852E-03
37	1.64652E+03	2.04142E-01	7.50208E-03
38	1.70454E+03	2.11336E-01	7.76645E-03
39	3.09439E+03	3.83656E-01	1.40991E-02
40	3.09605E+03	3.83861E-01	1.41066E-02
41	3.17747E+03	3.93957E-01	1.44776E-02
42	3.17819E+03	3.94046E-01	1.44809E-02

43	3.19220E+03	3.95783E-01	1.45447E-02
44	3.19503E+03	3.96133E-01	1.45576E-02
45	3.20325E+03	3.97153E-01	1.45951E-02
46	3.20359E+03	3.97195E-01	1.45966E-02
47	3.21950E+03	3.99168E-01	1.46691E-02
48	3.21995E+03	3.99223E-01	1.46712E-02

S3.3 Fulvene

Optimized geometry of fulvene, obtained as described in the main text.

#	atom	x [a.u.]	y [a.u.]	z [a.u.]
1	C	9.37362286E-02	8.38029269E-02	-2.13482432E+00
2	C	1.09360581E-01	2.18203574E+00	-6.76921660E-01
3	C	5.82926763E-02	1.39490208E+00	2.00116630E+00
4	C	1.11476607E-02	-1.39625278E+00	1.96138430E+00
5	C	3.14444015E-02	-2.16872151E+00	-4.74017869E-01
6	C	5.56576683E-02	2.90460878E+00	4.04866388E+00
7	H	1.20585072E-01	1.91509879E-02	-4.16142309E+00
8	H	1.51331973E-01	4.10953240E+00	-1.30305676E+00
9	H	-3.20689718E-02	-2.56423912E+00	3.61758192E+00
10	H	7.95099634E-03	-4.08566810E+00	-1.13490166E+00
11	H	9.33725178E-02	4.92893198E+00	3.87742492E+00
12	H	1.67350432E-02	2.14253957E+00	5.93184324E+00

Harmonic normal mode frequencies of fulvene, obtained as described in the main text.

mode	[cm^-1]	[eV]	[a.u.]
1	2.18229E+02	2.70570E-02	9.94325E-04
2	3.68012E+02	4.56277E-02	1.67679E-03
3	5.02379E+02	6.22871E-02	2.28901E-03
4	6.25980E+02	7.76116E-02	2.85217E-03
5	6.96602E+02	8.63677E-02	3.17395E-03
6	7.05650E+02	8.74895E-02	3.21518E-03
7	7.60422E+02	9.42803E-02	3.46474E-03
8	7.84157E+02	9.72231E-02	3.57288E-03
9	8.56995E+02	1.06254E-01	3.90476E-03
10	8.72245E+02	1.08145E-01	3.97424E-03
11	9.10741E+02	1.12918E-01	4.14964E-03
12	9.11737E+02	1.13041E-01	4.15418E-03
13	9.54594E+02	1.18355E-01	4.34945E-03
14	1.03605E+03	1.28453E-01	4.72058E-03
15	1.04750E+03	1.29873E-01	4.77275E-03
16	1.19056E+03	1.47611E-01	5.42460E-03
17	1.19240E+03	1.47839E-01	5.43300E-03
18	1.36583E+03	1.69341E-01	6.22317E-03
19	1.45922E+03	1.80921E-01	6.64871E-03
20	1.48325E+03	1.83899E-01	6.75817E-03
21	1.56669E+03	1.94245E-01	7.13838E-03
22	1.63617E+03	2.02859E-01	7.45492E-03
23	1.70087E+03	2.10881E-01	7.74973E-03
24	1.78082E+03	2.20793E-01	8.11400E-03
25	3.33309E+03	4.13251E-01	1.51867E-02
26	3.38860E+03	4.20134E-01	1.54396E-02
27	3.39916E+03	4.21442E-01	1.54877E-02
28	3.41028E+03	4.22821E-01	1.55384E-02
29	3.41245E+03	4.23090E-01	1.55482E-02
30	3.41956E+03	4.23971E-01	1.55806E-02

S3.4 Glycine

Optimized geometry of the I_p conformer of glycine, obtained as described in the main text.

#	atom	x [a.u.]	y [a.u.]	z [a.u.]
1	C	-8.21147284E-03	4.26694738E-01	-1.60269312E+00
2	O	-1.09944359E-02	-1.49848243E+00	-2.85537537E+00
3	O	-9.76435494E-03	2.77836774E+00	-2.62479310E+00
4	H	-1.40198663E-02	2.50373471E+00	-4.44536113E+00
5	C	-1.82500233E-03	5.30284348E-01	1.26985037E+00
6	H	1.66038754E+00	1.64194375E+00	1.86234844E+00
7	N	3.75992231E-03	-1.92113768E+00	2.51346205E+00
8	H	1.51758572E+00	-2.91285211E+00	1.82938563E+00
9	H	-1.51300595E+00	-2.91490364E+00	1.83888804E+00
10	H	-1.66431971E+00	1.63744410E+00	1.86952779E+00

Harmonic normal mode frequencies of the I_p conformer of glycine, obtained as described in the main text.

mode	[cm^-1]	[eV]	[a.u.]
1	7.17233E+01	8.89257E-03	3.26796E-04
2	2.48242E+02	3.07782E-02	1.13108E-03
3	2.66751E+02	3.30730E-02	1.21541E-03
4	4.72109E+02	5.85341E-02	2.15109E-03
5	5.18561E+02	6.42935E-02	2.36274E-03
6	6.39595E+02	7.92997E-02	2.91421E-03
7	6.69068E+02	8.29538E-02	3.04850E-03
8	8.58137E+02	1.06396E-01	3.90996E-03
9	9.28964E+02	1.15177E-01	4.23267E-03
10	9.99164E+02	1.23881E-01	4.55253E-03
11	1.16318E+03	1.44216E-01	5.29985E-03
12	1.18871E+03	1.47381E-01	5.41617E-03
13	1.20052E+03	1.48846E-01	5.46997E-03
14	1.32737E+03	1.64573E-01	6.04795E-03
15	1.40365E+03	1.74030E-01	6.39548E-03
16	1.43729E+03	1.78201E-01	6.54878E-03
17	1.46754E+03	1.81952E-01	6.68660E-03
18	1.66981E+03	2.07030E-01	7.60820E-03
19	1.85229E+03	2.29655E-01	8.43967E-03
20	3.09687E+03	3.83963E-01	1.41104E-02
21	3.16026E+03	3.91823E-01	1.43992E-02
22	3.51459E+03	4.35753E-01	1.60136E-02
23	3.59947E+03	4.46278E-01	1.64004E-02
24	3.77816E+03	4.68432E-01	1.72146E-02

References

(1) Granucci, G.; Persico, M.; Toniolo, A. Direct semiclassical simulation of photochemical processes with semiempirical wave functions. *The Journal of Chemical Physics* **2001**, *114*, 10608–10615.

(2) Plasser, F.; Granucci, G.; Pittner, J.; Barbatti, M.; Persico, M.; Lischka, H. Surface hopping dynamics using a locally diabatic formalism: Charge transfer in the ethylene dimer cation and excited state dynamics in the 2-pyridone dimer. *The Journal of Chemical Physics* **2012**, *137*, 22A514.

(3) González-Vázquez, J. sharcdyn/sharc_public: https://dx.doi.org/10.5281/zenodo.7352971. **2023**,
